# Key Roles of *CACNA1C*/Cav1.2 and CALB1/Calbindin in Prefrontal Neurons Altered in Cognitive Disorders

**DOI:** 10.1001/jamapsychiatry.2024.1112

**Published:** 2024-05-22

**Authors:** Dibyadeep Datta, Shengtao Yang, Mary Kate P. Joyce, Elizabeth Woo, Steven A. McCarroll, Guillermo Gonzalez-Burgos, Isabella Perone, Stacy Uchendu, Emi Ling, Melissa Goldman, Sabina Berretta, John Murray, Yury Morozov, Jon Arellano, Alvaro Duque, Pasko Rakic, Ryan O’Dell, Christopher H. van Dyck, David A. Lewis, Min Wang, Fenna M. Krienen, Amy F. T. Arnsten

**Affiliations:** 1 Department of Neuroscience, Yale University School of Medicine, New Haven, Connecticut; 2 Department of Psychiatry, Yale University School of Medicine, New Haven, Connecticut; 3 Department of Genetics, Harvard Medical School, Boston, Massachusetts; 4 Stanley Center for Psychiatric Research, Broad Institute of Massachusetts Institute of Technology and Harvard, Cambridge, Massachusetts; 5 Departments of Psychiatry and Neuroscience, University of Pittsburgh, Pittsburgh, Pennsylvania; 6 Basic Neuroscience Division, McLean Hospital, Belmont, Massachusetts; 7 Department of Psychiatry, Harvard Medical School, Boston, Massachusetts; 8 Princeton Neuroscience Institute, Princeton University, Princeton, New Jersey

## Abstract

**Importance:**

The risk of mental disorders is consistently associated with variants in *CACNA1C* (L-type calcium channel Cav1.2) but it is not known why these channels are critical to cognition, and whether they affect the layer III pyramidal cells in the dorsolateral prefrontal cortex that are especially vulnerable in cognitive disorders.

**Objective:**

To examine the molecular mechanisms expressed in layer III pyramidal cells in primate dorsolateral prefrontal cortices.

**Design, Setting, and Participants:**

The design included transcriptomic analyses from human and macaque dorsolateral prefrontal cortex, and connectivity, protein expression, physiology, and cognitive behavior in macaques. The research was performed in academic laboratories at Yale, Harvard, Princeton, and the University of Pittsburgh. As dorsolateral prefrontal cortex only exists in primates, the work evaluated humans and macaques.

**Main Outcomes and Measures:**

Outcome measures included transcriptomic signatures of human and macaque pyramidal cells, protein expression and interactions in layer III macaque pyramidal cells using light and electron microscopy, changes in neuronal firing during spatial working memory, and working memory performance following pharmacological treatments.

**Results:**

Layer III pyramidal cells in dorsolateral prefrontal cortex coexpress a constellation of calcium-related proteins, delineated by *CALB1* (calbindin), and high levels of *CACNA1C* (Cav1.2), *GRIN2B* (NMDA receptor GluN2B), and *KCNN3* (SK3 potassium channel), concentrated in dendritic spines near the calcium-storing smooth endoplasmic reticulum. L-type calcium channels influenced neuronal firing needed for working memory, where either blockade or increased drive by β1-adrenoceptors, reduced neuronal firing by a mean (SD) 37.3% (5.5%) or 40% (6.3%), respectively, the latter via SK potassium channel opening. An L-type calcium channel blocker or β1-adrenoceptor antagonist protected working memory from stress.

**Conclusions and Relevance:**

The layer III pyramidal cells in the dorsolateral prefrontal cortex especially vulnerable in cognitive disorders differentially express calbindin and a constellation of calcium-related proteins including L-type calcium channels Cav1.2 (*CACNA1C*), GluN2B-NMDA receptors (*GRIN2B*), and SK3 potassium channels (*KCNN3*), which influence memory-related neuronal firing. The finding that either inadequate or excessive L-type calcium channel activation reduced neuronal firing explains why either loss- or gain-of-function variants in *CACNA1C* were associated with increased risk of cognitive disorders. The selective expression of calbindin in these pyramidal cells highlights the importance of regulatory mechanisms in neurons with high calcium signaling, consistent with Alzheimer tau pathology emerging when calbindin is lost with age and/or inflammation.

## Key Points

**Question:**

Why do genetic risk studies of mental disorders find consistent associations with *CACNA1C*?

**Findings:**

In this study, the dorsolateral prefrontal cortical pyramidal cells most affected in cognitive disorders express elevated calcium-related signaling, delineated by *CALB1* (calcium-buffering protein calbindin), and high *CACNA1C* (L-type calcium channel [LTCC] Cav1.2), *GRIN2B* (NMDA receptor GluN2B), and *KCNN3* (SK3 channel) expression. These neurons require LTCC actions to sustain memory-related firing, but excessive levels, such as during stress, reduce firing via SK channel opening and induce pathology, especially when calbindin is lost with age and/or inflammation.

**Meaning:**

These data explain why both loss- and gain-of function variants in *CACNA1C* are associated with an increase in risk of cognitive disorders.

## Teaser

This study evaluates the role of *CACNA1C* variants in altered prefrontal neurons in cognitive disorders.

## Introduction

Genetic studies of mental disorders consistently find increased risk of cognitive deficits and mental illness with variants in *CACNA1C*, the gene that encodes the α1 subunit of the L-type calcium channel (LTCC) Cav1.2. Alterations in *CACNA1C* are associated with impaired working memory and function of the dorsolateral prefrontal cortex (dlPFC),^1,2^ and increased risk of schizophrenia, bipolar disorder, posttraumatic stress disorder, and Alzheimer disease (AD).^1,2,3,4,5,6,7^ All of these disorders are worsened by stress exposure^8,9^ and involve dysfunction of the recently evolved dlPFC, which subserves working memory, top-down control, and abstract reasoning.^10^ However, it is not known why this specific calcium channel is so important to dlPFC functioning. This question cannot be asked in mouse models, as rodents do not have a dlPFC. The current study addressed this key question in the dlPFC of humans and nonhuman primates and found that the most vulnerable neurons in dlPFC expressed a constellation of calcium-related proteins, including high levels of *CACNA1C*/Cav1.2, that render them especially vulnerable to dysfunction and neurodegeneration.

Pyramidal cells in layer III of the dlPFC are of particular interest, as this is the layer where reductions in spines and dendrites are most evident in schizophrenia,^11,12^ and where tau pathology and degeneration are prominent in AD.^13^ Layer III of the dlPFC in primates contains the pyramidal cell microcircuits that generate working memory and higher cognition through their recurrent excitatory connections on dendritic spines.^14^ These neurons are called delay cells, as they are able to sustain firing across the delay period in a working memory task, representing information in mind without sensory stimulation, the foundation of abstract thought.^15^ Delay cells rely on NMDA receptor GluN2B (*GRIN2B*) neurotransmission, the NMDA receptor that fluxes the highest levels of calcium to generate the persistent neuronal firing needed for working memory.^16^ In humans, GluN2B is associated with dlPFC function, schizophrenia, and AD.^17,18,19,20^ Importantly, the layer III dlPFC pyramidal cells that are most vulnerable to tau pathology and degeneration in AD express the calcium-buffering protein calbindin (encoded by *CALB1*) when young,^13^ as calbindin is a likely indicator that a neuron has high levels of calcium signaling. However, calbindin levels diminish with age and inflammation,^21,22,23^ leaving these neurons more vulnerable to calcium’s toxic effects.^24,25^

The current study performed transcriptomic analyses of pyramidal cells in the dlPFC of humans and macaques to learn why layer III *CALB1*-expressing pyramidal cells are especially vulnerable and to see if it relates to *CACNA1C* and calcium signaling. The molecules identified were then examined in more depth in macaques by examining the connections of these neurons, the interactions and locations of these calcium-related proteins within neurons, and their influence on neuronal firing and cognitive abilities. As Cav1.2 channels have an important role mediating the stress response in the heart, where β1-adrenoceptor (β1-AR) activation of Cav1.2 drives internal calcium release to potentiate cardiac output,^26^ we also examined the role of β1-AR–LTCC signaling in the loss of dlPFC function that occurs with exposure to uncontrollable stress, to help explain why *CACNA1C* variants are associated with deficits in dlPFC cognitive function in patients and why layer III pyramidal cells are especially susceptible to toxic insults.

## Methods

### Transcriptomic Data in the dlPFC of Human and Macaques

All research was conducted according to National Institutes of Health guidelines and approved by the Yale or University of Pittsburgh Institutional Animal Care and Use Committee (macaques) and the Partners Human Research Committee (humans). The human tissue was from a database from a recent study by Ling et al^27^; for further details, see the eMethods in Supplement 1.

Postmortem human dlPFC tissue from 50 neurotypical donors was processed for single-nucleus RNA sequencing (10× 3′ v3). Parallel studies were conducted with tissue from 2 adult female rhesus macaques. Multiple-label immunofluorescence and immuno-electron microscopy protein localization was performed in layer III of the dlPFC from adult rhesus macaque tissues using validated antibodies. Single-unit in vivo physiological recordings coupled with highly localized, iontophoretic drug application were performed in rhesus monkeys performing a spatial working memory task dependent on the dlPFC.

Exposure to uncontrollable stress,^28^ including the pharmacological stressor FG7142, impairs dlPFC working memory function in macaques^29^ and humans.^30^ FG7142 is a partial inverse agonist of the GABA_A_ receptor that induces a classic stress response in humans,^31^ monkeys, and rodents,^32^ including cortisol and corticosterone release^31,33,34^ and increased norepinephrine release in the prefrontal cortex.^35^ It can be used to assess stress humanely, using a dose that impairs accuracy but retains motivation to perform the task. The stress response was challenged with pretreatment with the β1-AR antagonist, betaxolol, or the LTCC antagonist nimodipine.

### Statistical Analyses

Repeated measures analyses of variance were used to assess drug effects on neuronal firing and behavioral performance. Two-tailed *P* values less than .05 were considered significant.

## Results

### Transcriptomic Analyses

**Figure 1.**
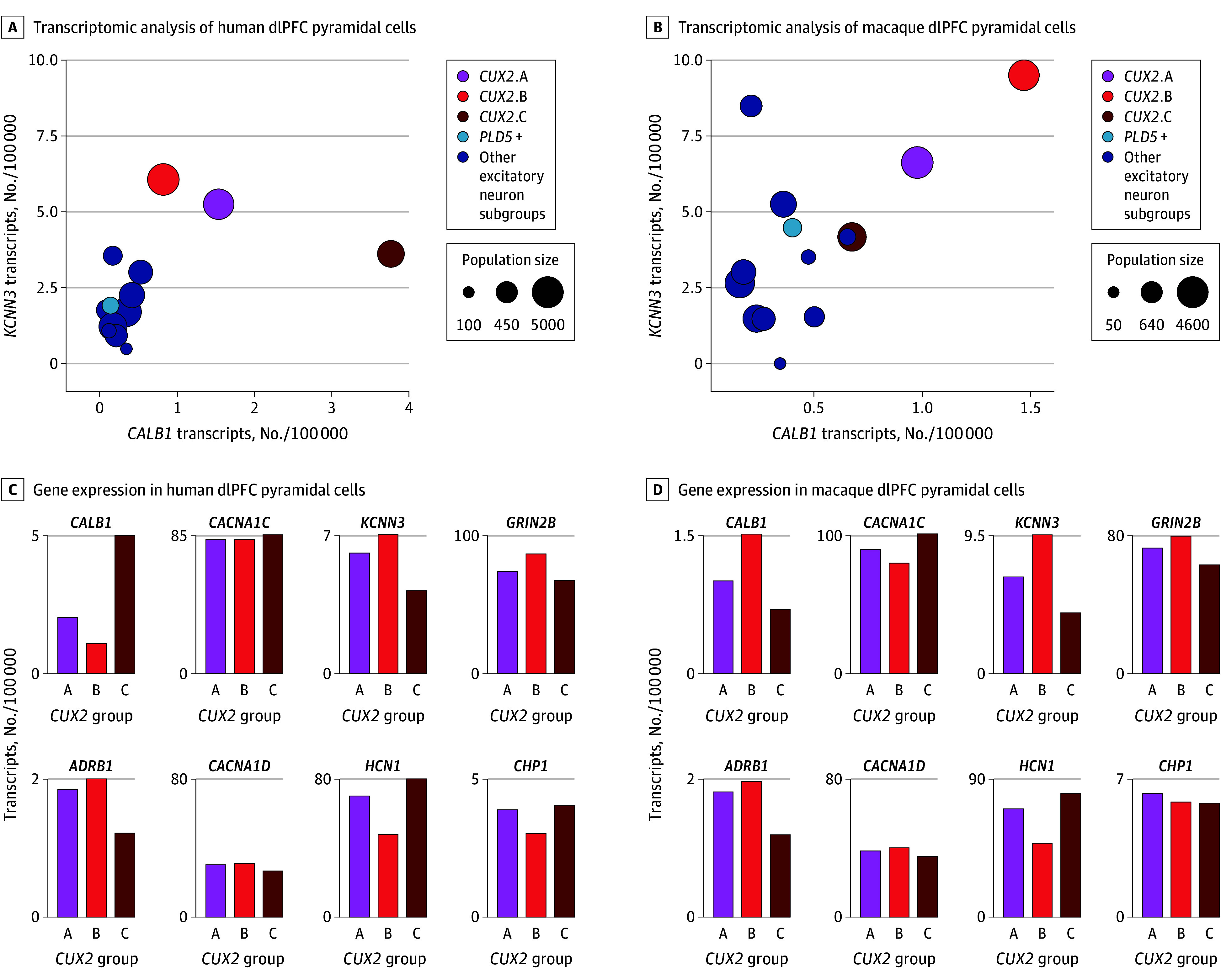
Transcriptomic Analyses of Excitatory Neurons in the Dorsolateral Prefrontal Cortex (dlPFC) of Humans and Macaques A, Transcriptomic analyses of human dlPFC pyramidal cells, showing expression levels of *CALB1* (encoding the calcium-binding protein calbindin) and *KCNN3* (encoding the SK potassium channel opened by calcium, SK3). Population sizes are expressed as number of nuclei out of 20 000. The *CUX2*-expressing cells have higher *CALB1* than other excitatory cells and very high levels of calcium-related genes, including *KCNN3*. B, Bar graph quantification of *CALB1*, *CACNA1C*, *KCNN3, GRIN2B* (encoding the GluN2B subunit of the NMDA receptor that fluxes high levels of calcium), *ADRB1* (encoding the β1-adrenoceptor [β1-AR], which drives Cav1.2 actions in the heart during stress exposure), *CACNA1D* (encoding the LTCC Cav1.3), *HCN1* (encoding the hyperpolarization-activated and cyclic nucleotide–gated channel opened by cyclic adenosine monophosphate), and *CHP1* (encoding the calcineurin inhibitor calcineurin homologous protein 1), in the 3 *CUX2* (*CUX2* A-C) dlPFC populations in the human dlPFC. C, Same as panel A but in the macaque dlPFC. D, Same as panel B but in the macaque dlPFC. Note lower levels of *CACNA1D* vs *CACNA1C* in both species. Expression values are normalized counts of the number of transcripts per 100 000 in each cell type.

Glutamatergic (*SLC17A7+*; encoding the vesicular glutamate transporter 1) pyramidal cells in the superficial layers of the dlPFC can be identified by their expression of the transcription factor *CUX2*. Single-nucleus RNA sequencing (eFigure 1A in Supplement 1) of the dlPFC in humans (Figure 1A and C) and macaques (Figure 1B and D) revealed 3 distinct subsets of *CUX2*-expressing pyramidal cells, which showed higher levels of *CALB1* than in other excitatory neurons (Figure 1A and B; eFigure 1B and C in Supplement 1; the data for calcium-related KEGG pathways across all pyramidal cell subgroups are shown in eTables 1 and 2 in Supplement 2). These cells expressed high levels of the LTCC Cav1.2 *CACNA1C*; *GRIN2B*, the NMDA receptor (NMDAR) with GluN2B subunits that fluxes the highest levels of calcium; *KCNN3*, the calcium-dependent SK3 potassium channel; and the calcineurin inhibitor *CHP1* encoding calcineurin homologous protein 1 (CHP1; Figure 1C and D). These patterns were especially distinct in human dlPFC neurons, where levels of *CACNA1C*, and especially *KCNN3* and *CHP1*, were generally higher than in other excitatory cell groups (Figure 1C; eFigure 1B in Supplement 1; eTables 1 and 2 in Supplement 2). They also expressed *ADRB1*, the β1-AR that drives cardiac Cav1.2 actions during stress (Figure 1C and D), and *HCN1*, encoding the hyperpolarization-activated and cyclic nucleotide–gated channel sensitive to cyclic adenosine monophosphate signaling (Figure 1). High levels of *KCNN3* are of particular interest, as the open state of these potassium channels is increased by calcium, causing reductions in neural firing.

We assessed the connectivity of *CALB1*-enriched dlPFC pyramidal cells in macaques, and confirmed their localization in layer III, by reanalyzing published data, where anatomical tracers had been used in tandem with laser-capture microdissection of layer III pyramidal cells.^36^ These layer III cells expressed high levels of *CUX2* as expected, and showed that *CALB1*-enriched cells preferentially projected to the contralateral dlPFC and not the ipsilateral parietal association cortex (1.4-fold enrichment in *CALB1* for contralaterally projecting). Although projections cannot be identified in the human brain, microarray data^37^ of laser-capture microdissected cells that compared human layer III vs layer V dlPFC pyramidal neurons found a 35.1-fold enrichment of *CUX2* and 8.5-fold enrichment of *CALB1* in layer III pyramidal neurons compared to layer V, consistent with *CALB1* enrichment in macaque layer III pyramidal cells.

### Protein Expression at the Cellular and Ultrastructural Levels

**Figure 2.**
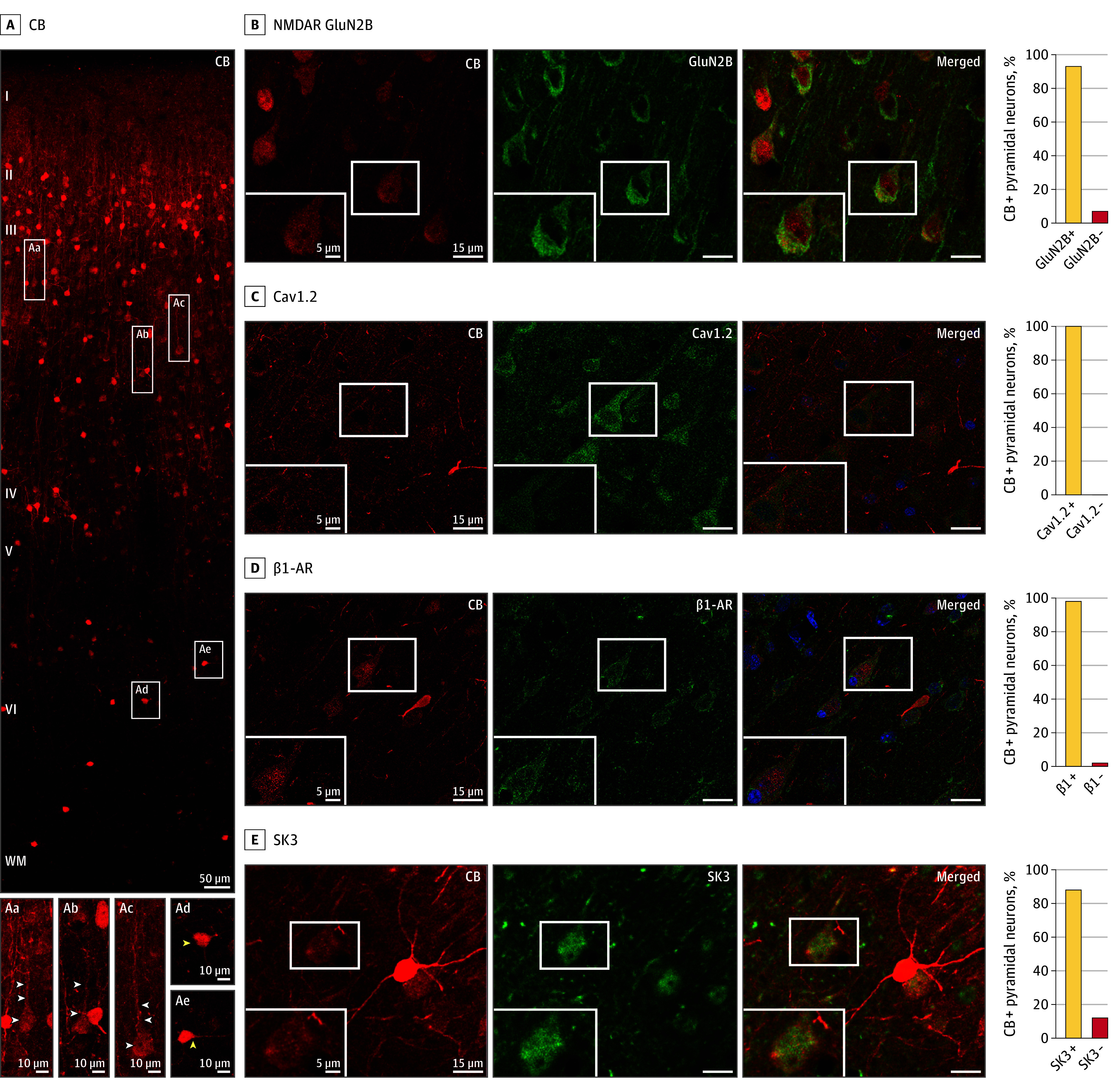
Calbindin-Expressing Pyramidal Cells in Layer III of the Dorsolateral Prefrontal Cortex (dlPFC) of Macaques and NMDA Receptor–GluN2B, Cav1.2 Channels, SK3 Channels, and β1-Adrenoceptor (β1-AR) A, Expression of the calcium-binding protein calbindin (CB), across the cortical column in the dlPFC of macaques. Pyramidal cells (examples in white rectangles) are concentrated in layer III and have modest calbindin expression (Aa-Ac; white arrowheads), while interneurons are throughout all layers, especially in layer II, and have intense calbindin expression (Ad-Ae; yellow arrowheads). B-E, Calbindin-expressing pyramidal cells coexpress the following proteins: B, NMDA receptor (NMDAR) GluN2B; C, Cav1.2 (amplified with biotin-streptavidin); D, β1-adrenoceptor (β1-AR) (amplified with biotin-streptavidin); and E, SK3. The intensely labeled, bright red neurons (eg, in E) are calbindin-expressing GABAergic interneurons, which have higher levels of calbindin than pyramidal cells. The percentage of calbindin-expressing pyramidal cells coexpressing each protein is shown on the right. Where present, blue labeling is the Hoechst nuclear counterstain.

The transcriptomic data indicate that calbindin-expressing layer III dlPFC pyramidal cells should coexpress Cav1.2, NMDAR GluN2B, and SK3 channels. This hypothesis was supported in layer III of the dlPFC of macaques using multiple-label immunofluorescence, where almost all calbindin-expressing pyramidal cells coexpressed these proteins (Figure 2B-E; eFigure 2 in Supplement 1). Most layer III pyramidal cells expressing Cav1.2 also expressed β1-AR (eFigure 2 in Supplement 1), similar to cardiac muscle.

**Figure 3.**
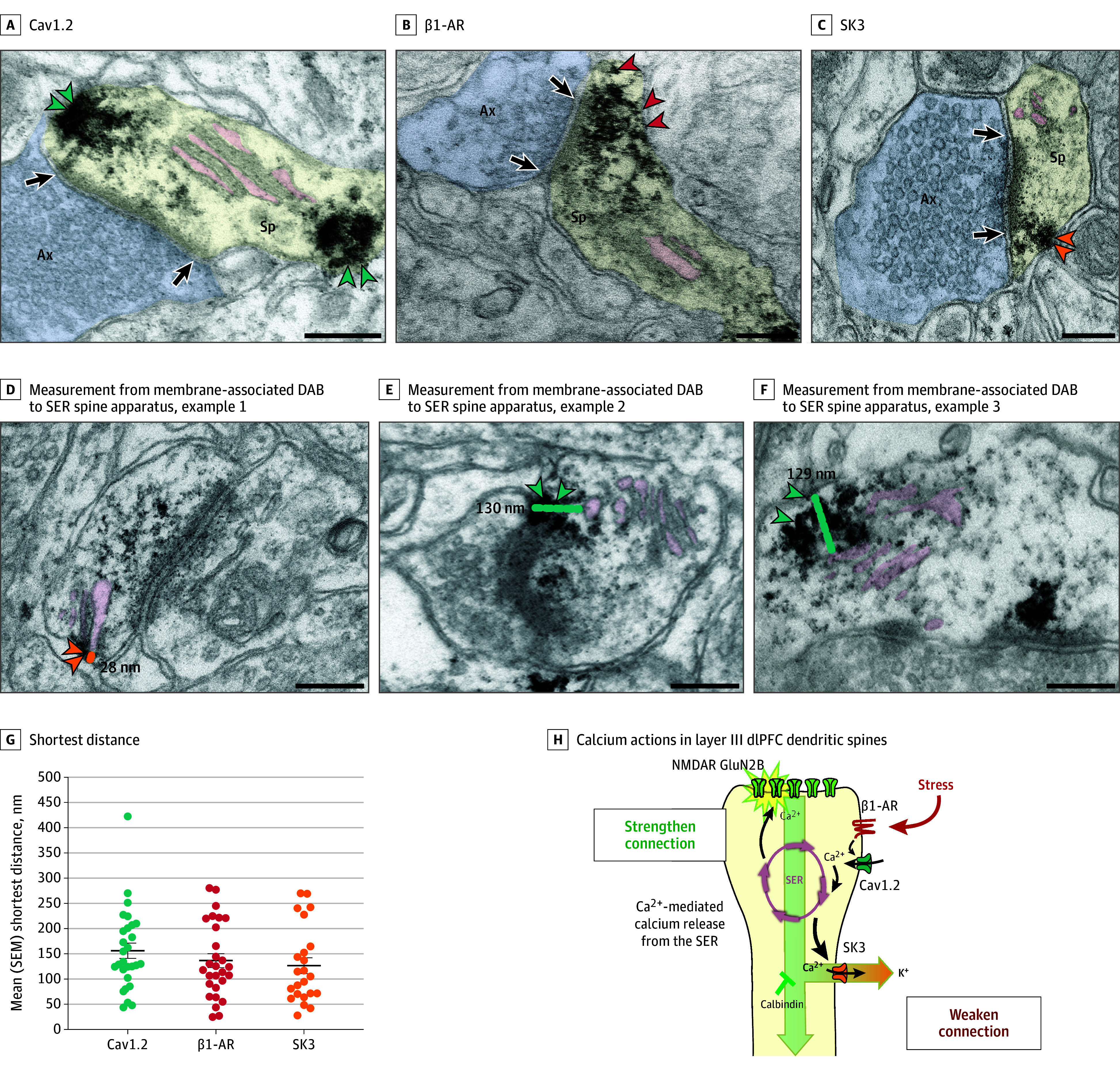
Immuno-Electron Microscopy Ultrastructural Localization of Cav1.2 Channels, SK3 Channels, and β1-Adrenoceptor (β1-AR) in Layer III of the Dorsolateral Prefrontal Cortex (dlPFC) in Macaques Cav1.2 (A, teal arrowheads), β1-AR (B, red arrowheads), and SK3 channels (C, orange arrowheadss) can be seen in dendritic spines receiving asymmetric (presumed glutamatergic) synapses, often localized on the plasma membrane near the calcium-storing and -releasing smooth endoplasmic reticulum (SER; termed the spine apparatus when it is elaborated in the dendritic spine). The SER spine apparatus is highlighted with pink pseudocoloring. Note that the Cav1.2 labeling can be seen near the postsynaptic density (PSD) and near the SER; additional examples can be seen in eFigures 3 and 4 in Supplement 1. Calbindin was not examined, as it is a cytosolic protein with diffuse labeling. D-F, Examples of measurements from center of membrane-associated diaminobenzidine (DAB) label to the SER spine apparatus. G, Shortest distance measured from center of membrane-associated DAB Cav1.2 (teal), β1-AR (red), and SK3 (orange) channel label to the SER spine apparatus; each dot represents a spine, and the black bar depicts the mean, with error bars indicating SEMs. Note that the DAB label may have obscured the SER spine apparatus in some instances; thus, the proteins on the plasma membrane may have been even closer than measured. H, A working model of calcium actions in layer III dlPFC dendritic spines, showing a functional calcium-related interactome. Under nonstress conditions, moderate Cav1.2 L-type voltage-gated Ca2+ channel actions, including potential calcium-mediated calcium release through ryanodine receptors (RYR) on the SER spine apparatus, are needed for strong working memory delay-related firing, possibly by depolarizing the postsynaptic density (PSD) to permit NMDA receptor (NMDAR) neurotransmission. Previous research has shown that the PSD contains NMDA receptors with GluN2B subunits, and that delay cell firing during working memory depends on NMDA receptor–GluN2B neurotransmission. Under stressful conditions, high levels of norepinephrine stimulate β1-AR to activate a large number of L-type calcium channel (LTCC)/Cav1.2 calcium channels. This may induce high levels of calcium-mediated calcium release from the SER, as occurs with the stress response in the heart. High levels of calcium would open large numbers of SK potassium (K^+^) channels, rapidly reducing neuronal firing. Feedforward, calcium–cyclic adenosine monophosphate signaling would also open cyclic adenosine monophosphate–sensitive channels on spines (eg, hyperpolarization-activated and cyclic nucleotide–gated slack [K^+^]) channels. As dlPFC delay cell firing is needed for working memory, these intracellular signaling events lead to cognitive impairment. Sustained reductions in neuronal firing or high levels of cytosolic calcium would also lead to degeneration, especially when the protective effects of calbindin are lost with age and/or inflammation. Black arrows indicate synapses. Ax indicates axon terminal (pseudocolored blue); Mit, mitochondria; Sp, spine (pseudocolored yellow). Scale bars = 200 nm.

Immuno-electron microscopy was used to observe the location of these proteins within layer III dlPFC pyramidal cells. Previous research documented NMDAR GluN2B on layer III dendritic spines within the postsynaptic density.^16^ Here we found that Cav1.2, SK3 channels, and β1-AR were also concentrated in layer III dlPFC spines, as well as some expression on dendrites and glia (Figure 3A-C; eFigures 3-6 in Supplement 1). Cav1.2 channels were often within or near the postsynaptic density (eg, Figure 3A and E; eFigures 3A and 4A and D in Supplement 1), and were often found in the membrane near the calcium-storing and -releasing smooth endoplasmic reticulum, occasionally in extremely close proximity (eg, SK3 = 28 nm; Cav1.2 = 44 nm) (Figure 3D-G). This is similar to cardiac muscle, where Cav1.2 drives internal calcium release.^38^ Thus, Cav1.2 may play a comparable role in layer III dlPFC spines (schematically illustrated in Figure 3H).

### Effects on dlPFC Delay Cell Firing During Working Memory

It is already known that delay cell firing is dependent on NMDAR neurotransmission, including NMDAR with GluN2B subunits.^16^ However, the roles of Cav1.2, β1-AR, and SK channels on delay cell firing have not been known. Here we coupled iontophoresis for local drug delivery with single-unit recordings in older macaques performing the oculomotor delayed response test of spatial working memory, where the monkey must remember a spatial location over a delay period of several seconds and then make an eye movement to the remembered location for a juice reward (eFigures 7 and 8 in Supplement 1; see legend to eFigure 7A in Supplement 1 for a description of the task). Middle-aged (12-16 years) and aged (≥17 years) macaques have disinhibited cyclic adenosine monophosphate–calcium signaling in the dlPFC^39^ and thus are especially helpful for testing the effects of LTCC channel blockade. There are no compounds selective for Cav1.2 or for the SK3 channel isoform; thus, this work used LTCC compounds that target both Cav1.2 and Cav1.3, and an SK channel blocker that targets all SK isoforms. The transcriptomic data showed that there were much lower levels of *CACNA1D* (Cav1.3) than *CACNA1C* in the *CALB1*-expressing pyramidal cells (Figure 1C and D), and Cav1.3 had a broader distribution on neurons than Cav1.2, including expression on presynaptic terminals (eFigure 9 in Supplement 1). However, Cav1.3 had effects similar to Cav1.2 in many locations^40^ and may contribute to effects on delay cell firing.

**Figure 4.**
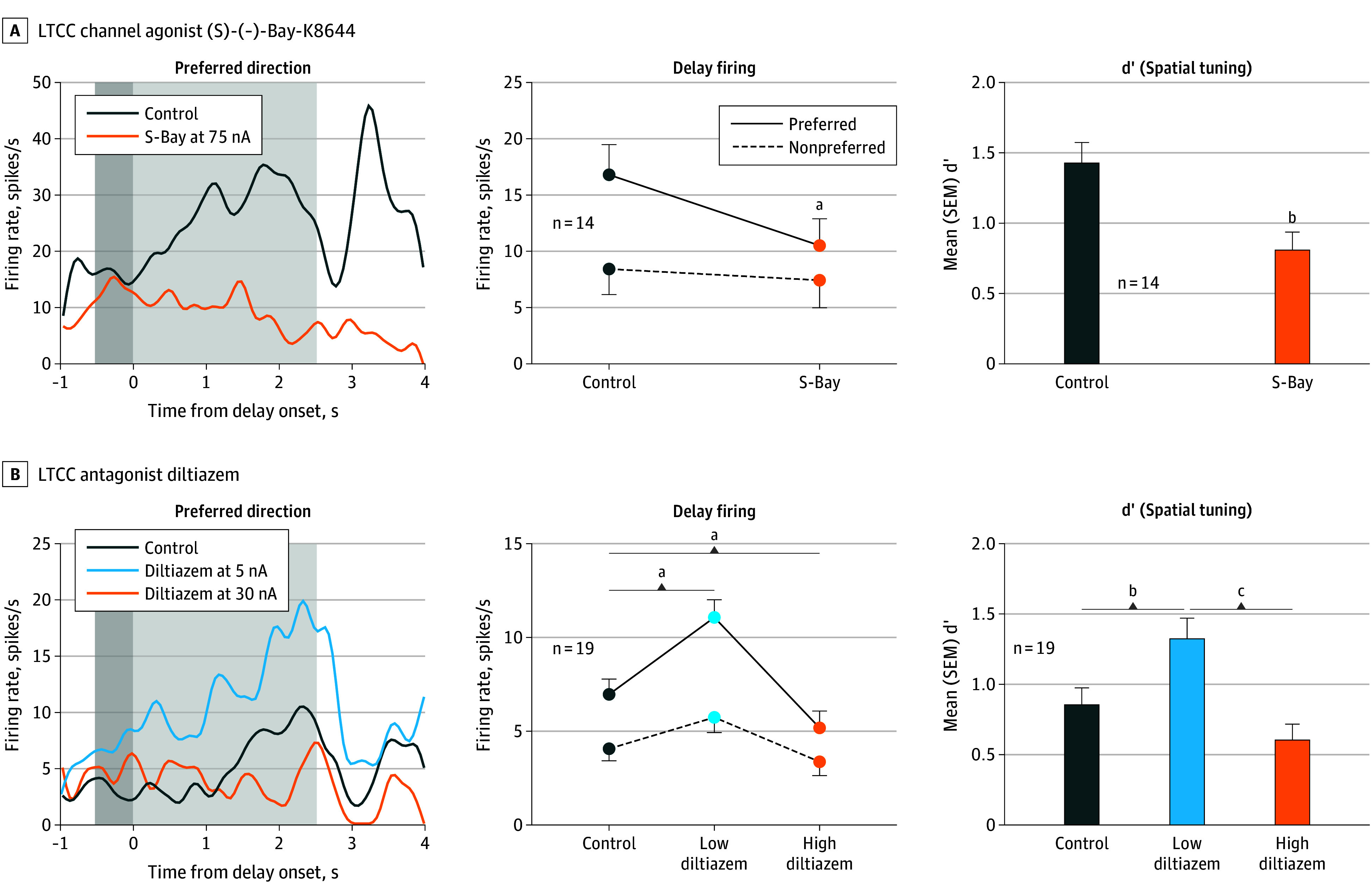
Recordings From the Dorsolateral Prefrontal Cortex (dlPFC) in Macaques During Working Memory: the Effects of Manipulating LTCC Activity on Delay Cell Firing in the dlPFC of Macaques The left subpanels show an example neuron’s delay-related firing under control and drug conditions with dark gray indicating the cue period and light gray indicating the delay period; the middle subpanels show the mean (SEM) delay-related firing for all delay cells under control vs drug conditions; the right subpanels show the mean (SEM) d’ measure of spatial tuning for all delay cells under control vs drug conditions. A, The LTCC channel agonist, (S)-(-)-Bay-K8644 (S-Bay; orange), was associated with reduced delay-related firing (*R* 2-way analysis of variance, *F*_1,13_ = 20.06; *P* < .001) and decreased d’ (paired *t* test, *t*_13_ = 4.16; *P* = .001). B, The LTCC antagonist diltiazem (dil) produced an inverted-U dose-response, with low doses of diltiazem (5-20nA, light blue) increasing delay-related firing and spatial tuning, while high doses (30-50nA, orange) were associated with reduced delay firing and spatial tuning (firing rate: *R* 2-way analysis of variance, *F*_2,26_ = 26.90, *P* < .001; spatial tuning: *R* 1-way analysis of variance, *F*_1.786,32.15_ = 13.86, *P* < .001). ^a^*P* < .0001. ^b^*P* < .01. ^c^*P* < .005.

**Figure 5.**
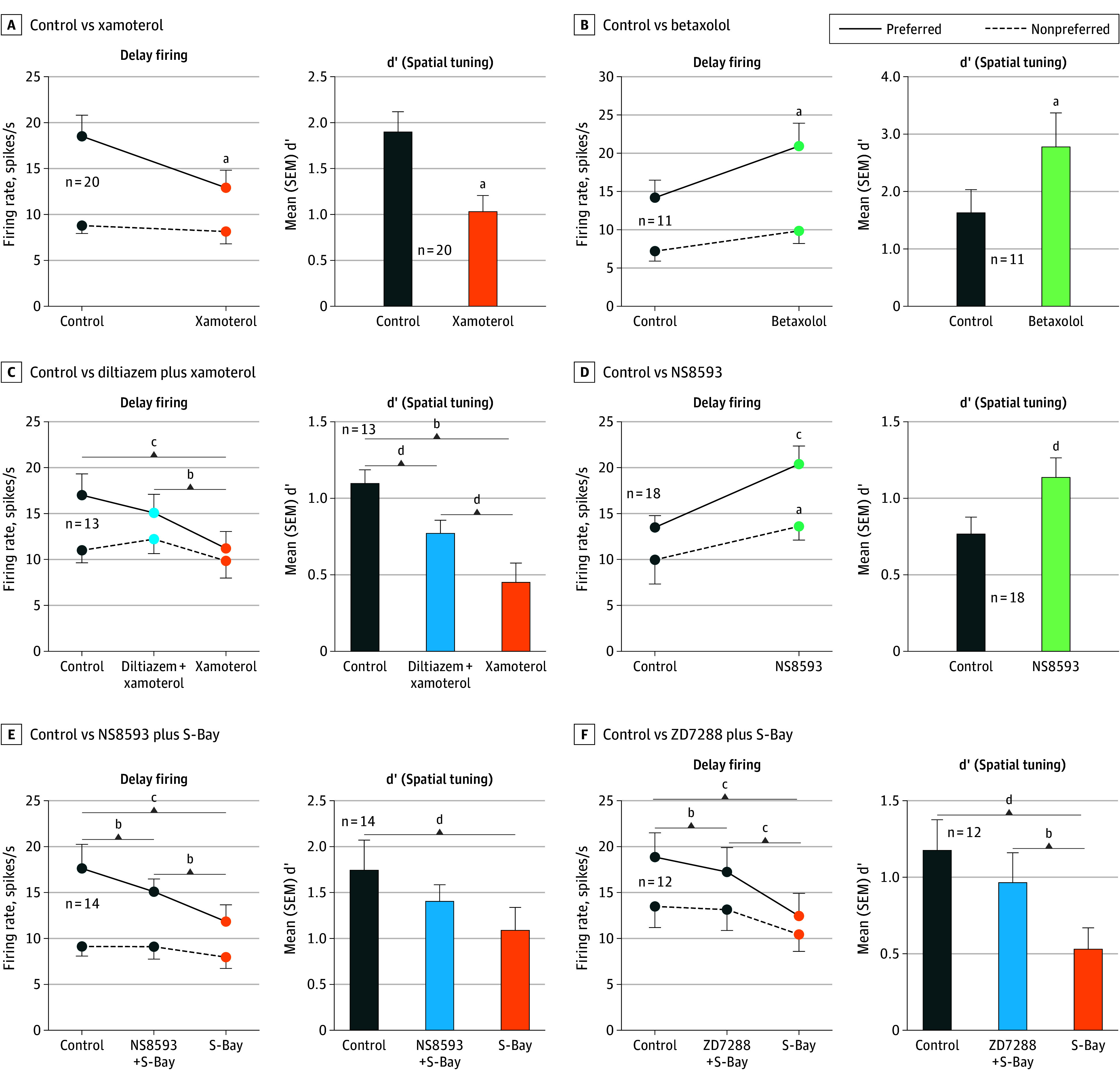
Roles of β1-Adrenoceptor (β1-AR), L-Type Calcium Channel (LTCC), and SK Channels in Dorsolateral Prefrontal Cortex (dlPFC) Delay Cell Firing The left subpanels show the mean (SEM) delay-related firing for all delay cells under control vs drug conditions; the right subpanels show the mean (SEM) d’ measure of spatial tuning for all delay cells under control vs drug conditions. A, The β1-AR agonist xamoterol (orange) was associated with reduced delay-related firing of dlPFC delay cells for the neurons’ preferred direction but not for nonpreferred directions (*R* 2-way analysis of variance, *F*_1,19_ = 12.99; *P* = .002), leading to a significant reduction in the d’ measure of spatial tuning (paired *t* test, *t*_19_ = 4.68; *P* < .001). B, Conversely, the β1-AR antagonist betaxolol (green) was associated with enhanced delay-related firing (*R* 2-way analysis of variance, *F*_1,10_ = 6.42; *P* = .03), and increased d’ measures of spatial tuning (paired *t* test, *t*_10_ = 3.36; *P* = .007). C, The reducing effects of the β1-AR agonist xamoterol were blocked by pretreatment with the LTCC antagonist, diltiazem (*R* 2-way analysis of variance, *F*_2,24_ = 5.90; *P* = .008; Tukey multiple comparisons: preferred direction, control vs diltiazem plus xamoterol; *P* = .16; control vs xamoterol, *P* < .001; diltiazem plus xamoterol vs xamoterol, *P* = .002). D, The SK channel blocker NS8593 was significantly associated with increased delay firing for the neurons’ preferred direction, as well as a smaller increase for nonpreferred directions (*R* 2-way analysis of variance, *F*_1,17_ = 12.13; *P* = .003), leading to a significant increase in d’ measure of spatial tuning (paired *t* test, *t*_17_ = 2.18; *P* = .04). E, The mean firing rate of 14 dlPFC delay cells, showing that the reducing effects of the LTCC agonist S-Bay were blocked by pretreatment with the SK channel antagonist NS8593 (*R* 2-way analysis of variance, *F*_2,26_ = 5.89; *P* = .008). F, The mean firing rate of 12 dlPFC delay cells, showing that the reducing effects of the LTCC agonist S-Bay were blocked by pretreatment with the hyperpolarization-activated and cyclic nucleotide–gated channel antagonist ZD7288 (*R* 2-way analysis of variance, *F*_2,22_ = 13.47; *P* < .001). ^a^*P* < .001. ^b^*P* < .005. ^c^*P* < .0001. ^d^*P* < .05.

We found a narrow inverted-U dose response, where either inadequate (extensive LTCC blockade with high-dose diltiazem) or excessive (LTCC activation with S-Bay-K8644) LTCC actions caused a loss of delay-related neuronal firing, with low-dose LTCC blockade actually enhancing delay cell firing (Figure 4A and B; eFigures 10A and 11A in Supplement 1). Further experiments tested the hypothesis that, as in muscle, β1-AR may drive LTCC actions. We found that stimulation of β1-AR with xamoterol, like LTCC channel opening, markedly reduced delay cell firing, while the β1-AR antagonist betaxolol enhanced firing, and that the detrimental effects of β1-AR could be prevented or reversed with LTCC blockade (Figure 5A-C; eFigures 10B, 11B, and 12A and B in Supplement 1). The loss of neuronal firing involved the opening of SK potassium channels, since blockade of SK channels with NS8593 enhanced delay cell firing (Figure 5D; eFigure 13A in Supplement 1) and reduced the detrimental effects of LTCC opening (Figure 5E; eFigure 13B in Supplement 1). Hyperpolarization-activated and cyclic nucleotide–gated channel blockade with ZD7288 also protected against excessive LTCC activation (Figure 5F; eFigure 13C in Supplement 1). These channels are expressed in *CALB1*-expressing pyramidal cells (Figure 1), and localize on layer III dlPFC spines.^41^ They are opened by cyclic adenosine monophosphate signaling and can couple with slack potassium channels,^42^ consistent with feedforward calcium–cyclic adenosine monophosphate signaling in layer III spines.

### Cognitive Behavior

The physiological data suggest that β1-AR activation of LTCC may contribute to dlPFC dysfunction during uncontrollable stress. This hypothesis was tested in 6 rhesus macaques performing the delayed response spatial working memory task, using a low dose of a pharmacological stressor, FG7142, that impairs accuracy (*F*_1,5_ = 49.0; *P* < .001 compared to vehicle; eFigure 14A in Supplement 1; *F*_1,6_ = 40.4; *P* < .001 compared to vehicle; eFigure 14B in Supplement 1) but allows completion of the task. Pretreatment with a dose of the β1-AR antagonist, betaxolol, or of the LTCC blocker, nimodipine, protected working memory performance from the detrimental effects of mild stress (*F*_1,5_ = 29.1; *P* = .003 compared to vehicle + stress; eFigure 14A in Supplement 1; *F*_1,6_ = 25.8; *P* = .002 compared to vehicle + stress; eFigure 14B in Supplement 1). These data are consistent with excessive Cav1.2 actions impairing dlPFC cognitive function.

## Discussion

This study identified a constellation of calcium-related signaling proteins in the layer III pyramidal cells in the dlPFC known to be most vulnerable in cognitive disorders. We found that, in the dlPFC of both humans and macaques, these cells had an especially high expression of *CALB1*, as well as *CACNA1C*, *GRIN2B*, *KCNN3*, and *CHP1*, encoding the calcium-buffering protein, calbindin, the LTCC Cav1.2 channel, the NMDAR GluN2B that fluxes high levels of calcium into the neuron, the SK3 potassium channel whose activation is increased by calcium, and the calcineurin inhibitor CHP1, respectively. Additional macaque data showed that the layer III pyramidal cells that project to the contralateral dlPFC were enriched in *CALB1*, suggesting that increased calcium may be needed to maintain firing across the corpus callosum to integrate working memory across the hemispheres.^43^ Immuno-electron microscopy demonstrated that Cav1.2, SK3 channels, and β1-AR were all concentrated on layer III dendritic spines, similar to NMDAR GluN2B,^16^ with Cav1.2 on the plasma membrane near the calcium-storing spine apparatus, positioned to further increase calcium actions via internal release. Physiological recordings from cognitively engaged macaques showed that either inadequate or excessive LTCC actions reduced delay cell firing, with excessive signaling driven by β1-AR stimulation and the opening of SK potassium channels. Comparable effects were seen at the behavioral level, with stress-induced working memory impairment rescued by LTCC or β1-AR blockade. These data reveal a powerful mechanism by which stress impairs dlPFC cognitive function, and also suggest that either loss-of-function or gain-of-function variants in *CACNA1C* would be harmful to dlPFC function and increase risk of neuropsychiatric disorders. As LTCCs are often found to have only excitatory effects on neuronal firing,^44^ the loss of dlPFC neuronal firing with excessive LTCC actions via SK potassium channel opening is particularly noteworthy, especially as *KCNN3* SK3 channels are preferentially enriched in dlPFC *CALB1*-expressing excitatory cells in the human dlPFC. Thus, these cells may be especially vulnerable to loss of firing under conditions of high calcium. The high levels of the calcineurin inhibitor CHP1 may also make these cells more vulnerable to tau pathology, as calcineurin dephosphorylates tau. Thus, the current data help to explain why variants that either decrease or increase *CACNA1C* actions would be associated with prefrontal cortex dysfunction and thus increased risk of mental disorders, such as schizophrenia, and why loss of calbindin from these pyramidal cells with age and/or inflammation would contribute to neuropathology in AD, where elevated cytosolic calcium is known to contribute to tau pathology and neurodegeneration.^24^ These findings are a rare example where transcriptomic and genomic data can be related to the dysfunction of a higher cortical circuit, illuminating how molecular insults give rise to symptoms of cognitive impairment.

It should be noted that the syndromes associated with variants in *CACNA1C* are complex, for example with a variety of single-nucleotide variants causing variations of Timothy syndrome, a multisystem disorder typified by cardiac abnormalities, which sometimes includes neurodevelopmental cognitive delays relevant to the current data.^45^ How specific *CACNA1C* variants alter channel function, and the functional ramifications of these alterations to cardiac and neural physiology, is an area of ongoing and future research, with the current data highlighting the importance of these channels to the cognitive functions of the dlPFC.

### Limitations

An important caveat of the current research is the lack of pharmacological agents to dissect Cav1.2 from potential Cav1.3 (*CACNA1D*) actions. It is likely that excessive opening of both LTCC subtypes contribute to the loss of delay cell firing and working memory abilities, as Cav1.3 shares many properties with Cav1.2, including interacting with ryanodine receptors on the smooth endoplasmic reticulum to increase internal calcium release.^40^ However, the predominant expression of *CACNA1C* compared to *CACNA1D* in these dlPFC pyramidal cells, and their concentration on dendritic spines, suggests that Cav1.2 may be the more important isoform in dlPFC-related clinical disorders.

Figure 3H presents a working model based on the current and previous immuno-electron microscopy and physiology data, focusing on calcium actions in layer III dlPFC dendritic spines. It is already known that NMDAR GluN2B (*GRIN2B*) neurotransmission is crucial at these synapses,^16^ consistent with current findings. The current study also found a concentration of LTCC Cav1.2 on spines near the smooth endoplasmic reticulum spine apparatus, where we hypothesize that calcium influx through LTCCs could drive additional internal calcium release, as occurs in muscle. Moderate amounts of calcium near the postsynaptic density may facilitate NMDAR neurotransmission and support recurrent firing across the hemisphere, as high-dose LTCC blockade reduced delay cell firing. However, high levels of Cav1.2 opening, such as those driven by NE β1-AR actions during stress, reduced delay cell firing via opening of SK channels. As norepinephrine has low affinity for β1-AR,^46^ it would require high levels of norepinephrine release, as occurs with stress, to engage this mechanism, similar to the heart, where the fight or flight response involves β1-AR activation of Cav1.2 inducing calcium-mediated calcium release from the sarcoplasmic reticulum to increase muscle contraction.^26^ Although it is not technically feasible to test this hypothesis in dlPFC spines, the proximity of both Cav1.2 and SK3 channels to the smooth endoplasmic reticulum in spines suggests similar actions may occur in layer III of the dlPFC. This working model is consistent with human data showing that cognitive operations dependent on the dlPFC are impaired by stress exposure via β-AR stimulation.^47^ As stress exposure is a risk factor for multiple neuropsychiatric disorders, including schizophrenia^48^ and AD,^9,49,50^ this working model may help to explain why increased risk of neuropsychiatric disorders is consistently associated with alterations in *CACNA1C*.

The dlPFC is profoundly altered in schizophrenia, such as with a reduced dlPFC blood oxygen level–dependent response during working memory highly correlating with symptoms of thought disorder^51^ and shorter dendrites and fewer spines preferentially in layer III, with intact spines in deep layers.^11^ Alterations in *CACNA1C* are consistently associated with an increased risk of schizophrenia, as well as other neuropsychiatric disorders typified by prefrontal cortex dysfunction.^7^ Variants in *CACNA1C* are numerous, and their impact on channel function is complex and a topic of current research.^52^ Current data suggest that the impact of a variant may depend on where the channel is expressed, with distinct molecular interactions in a tissue-specific manner.^52^ However, several alterations associated with schizophrenia and impaired dlPFC function appear to be gain-of-function variants,^1,53,54^ especially when LTCC blockade can normalize dlPFC activity in these individuals.^55^ The current data show that a gain in LTCC Cav1.2 actions would magnify stress-induced prefrontal cortex dysfunction, resulting in a loss of neuronal firing. As both loss of neuronal firing and/or sustained increases in calcium signaling can induce dendritic atrophy,^25^ this mechanism could also contribute to the selective changes in spines and dendrites in layer III of the dlPFC in schizophrenia.^11^ It is not known if the layer III dlPFC pyramidal cells with altered dendrites in schizophrenia express calbindin, prior to descent into illness for instance, an important area for future research. Risk of schizophrenia has also been associated with variants in *GRIN2B*,^56^ although alterations in this receptor are more often associated with more general neurodevelopmental intellectual impairment, consistent with the key roles of this receptor in neuronal and circuit development.^57^ It is likely that alterations in *CACNA1C* also contribute to neurodevelopmental insults,^58^ including possible alterations in dendritic morphology^59^ and/or interneuron migration.^60^ However, the current data emphasize that altered *CACNA1C* LTCC function can also impact higher cognitive abilities in the adult dlPFC.

AD pathology in the dlPFC is associated with impaired executive functioning, working memory and abstract reasoning, and psychosis.^61,62,63^ Postmortem studies have shown that tau pathology and degeneration especially targets layer III dlPFC calbindin-expressing pyramidal cells but not interneurons.^13^ Calbindin/*CALB1* is lost from the dlPFC with age and inflammation in macaques and humans^21,22,23^; calbindin loss is selective to dlPFC pyramidal cells and related to the rise in tau hyperphosphorylation.^21^ The current data show that calbindin-expressing pyramidal cells in the dlPFC express magnified calcium signaling that would make them more vulnerable to AD pathology when calbindin’s protective effects are lost with age, especially within the context of elevated CHP1 inhibition of calcineurin-mediated dephosphorylation. Although there are some genetic links between calcium channels and sporadic AD (*CACNA1C* × *RYR3*^3^), it is likely that inflammatory insults to calbindin over a long life are a more common contributor to AD risk in this subset of pyramidal cells.

Another study^64^ found that the most vulnerable cells in AD entorhinal cortex express *RORB*. Thus, we examined *RORB* expression in the *CUX2A* and *CUX2C* dlPFC subgroups and found they also coexpressed *RORB* (eFigure 15 in Supplement 1). The current transcriptomic data also revealed a small subgroup of pyramidal cells (*PLD5^+^*) with extremely high *CACNA1C* and *GRIN2B* but little *CALB1*, *CUX2*, or *RORB* in both humans and macaques. Future research could discern whether this subgroup corresponds to the smaller number of pyramidal cells that develop tau pathology in layer Va of the dlPFC,^65^ a sublayer that also has extensive local recurrent connections in the dlPFC of macaques.^66^

## Conclusions

The findings of this study indicate that the layer III dlPFC pyramidal cells most vulnerable to pathology expressed a constellation of functionally interacting, calcium-related proteins, distinguished by *CALB1* (calbindin), and including high levels of LTCC Cav1.2 encoded by *CACNA1C*, *GRIN2B* encoding GluN2B NMDAR, and* KCNN3*, encoding an SK potassium channel that causes decreased neuronal firing under conditions of high calcium, localized near the calcium-storing smooth endoplasmic reticulum in dendritic spines. LTCC opening is needed to sustain neuronal firing, but high levels, such as with stress, reduce neuronal firing and impair working memory, helping to explain why both loss- and gain-of-function variants in *CACNA1C* were associated with impaired cognition and increased risk of mental disorders. As toxic levels of calcium are known to contribute to atrophy,^25^ and to tau and amyloid pathology over a long time frame,^24^ these neurons with increased calcium signaling would be at risk of neuropathology when the calcium-buffering effects of calbindin are lost with age and/or inflammation. Protecting these neurons would be a helpful strategy to maintain healthy cognitive function.

## Notes

Supplement 1. eFigure 1. Schematic of methods used to perform single nucleus RNAseq from human dlPFC and a 3D portrayal of CALB1, CACNA1C and GRIN2B expression in the excitatory neurons of human and macaque dlPFC eFigure 2. Additional examples of multiple label immunofluorescence (MLIF) of Cav1.2, β1-AR, NMDAR-GluN2B, or SK3 and calbindin co-expression in macaque layer III dlPFC pyramidal cells eFigure 3. Additional examples of immunoEM ultrastructural localization of Cav1.2 channels, SK3 channels and β1-AR in layer III macaque dlPFC eFigure 4. Cav1.2 LTCCs and β1-ARs are expressed in dendritic spines near the SER spine apparatus in layer III macaque dlPFC eFigure 5. Cav1.2 LTCCs and β1-ARs are expressed in pyramidal cell-like dendritic shafts in layer III macaque dlPFC eFigure 6. SK3 channels are expressed on the plasma membrane of macaque layer III dlPFC spines and dendrites, and in astrocytes, but not in axons eFigure 7. Schematic of methods used for single unit recording from macaque dlPFC during a spatial working memory task, with iontophoretic delivery of drug to alter the local neurochemical environment eFigure 8. Estimate of drug concentration from iontophoresis at 20nA at 100 μm from electrode tip eFigure 9. Cav1.3 LTCCs have a more generalized expression pattern in macaque layer III dlPFC, including axonal labeling eFigure 10. Iontophoresis of the LTCC agonist, S-Bay K 6844, or the β1-AR agonist, xamoterol, markedly reduced delay-related firing and spatial tuning of dlPFC Delay cells eFigure 11. Iontophoresis of the LTCC antagonist, diltiazem, produced an inverted-U dose response, while the β1-AR antagonist, betaxolol, markedly enhanced delay-related firing and spatial tuning of dlPFC Delay cells eFigure 12. Blocking LTCCs prevents/reverses the reduction in firing from β1-AR stimulation eFigure 13. Iontophoresis of the SK channel blocker, NS8593, markedly enhanced the delay-related firing and spatial tuning of dlPFC Delay cells, while blockade of either SK or HCN channels blocked the effects of LTCC opening eFigure 14. Behavioral data showing performance of the spatial delayed response test of spatial working memory under control vs. stress conditions, with pretreatment with vehicle or test drug eFigure 15. RORB is widely expressed in dlPFC pyramidal cells, including in the CUX2A and CUX2C subgroups eReferences

Supplement 2. eTable 1. Transcript levels of excitatory cell groups in human dlPFC (values represent log10 values of those in Figure 1) eTable 2. Transcript levels of excitatory cell groups in macaque dlPFC (values represent log10 values of those in Figure 1)

Supplement 3. Data sharing statement

## References

[1] Bigos KL, Mattay VS, Callicott JH, . Genetic variation in *CACNA1C* affects brain circuitries related to mental illness. Arch Gen Psychiatry. 2010;67(9):939-945. doi:10.1001/archgenpsychiatry.2010.96 20819988 PMC3282053

[2] Cosgrove D, Mothersill O, Kendall K, ; Wellcome Trust Case Control Consortium. Cognitive characterization of schizophrenia risk variants involved in synaptic transmission: evidence of *CACNA1C*’s role in working memory. Neuropsychopharmacology. 2017;42(13):2612-2622. doi:10.1038/npp.2017.123 28607492 PMC5686488

[3] Koran ME, Hohman TJ, Thornton-Wells TA. Genetic interactions found between calcium channel genes modulate amyloid load measured by positron emission tomography. Hum Genet. 2014;133(1):85-93. doi:10.1007/s00439-013-1354-8 24026422 PMC4045094

[4] Krzyzewska IM, Ensink JBM, Nawijn L, . Genetic variant in *CACNA1C* is associated with PTSD in traumatized police officers. Eur J Hum Genet. 2018;26(2):247-257. doi:10.1038/s41431-017-0059-1 29362489 PMC5838973

[5] Gordovez FJA, McMahon FJ. The genetics of bipolar disorder. Mol Psychiatry. 2020;25(3):544-559. doi:10.1038/s41380-019-0634-7 31907381

[6] Trubetskoy V, Pardiñas AF, Qi T, ; Indonesia Schizophrenia Consortium; PsychENCODE; Psychosis Endophenotypes International Consortium; SynGO Consortium; Schizophrenia Working Group of the Psychiatric Genomics Consortium. Mapping genomic loci implicates genes and synaptic biology in schizophrenia. Nature. 2022;604(7906):502-508. doi:10.1038/s41586-022-04434-5 35396580 PMC9392466

[7] Harrison PJ, Husain SM, Lee H, . *CACNA1C* (Ca_V_1.2) and other L-type calcium channels in the pathophysiology and treatment of psychiatric disorders: advances from functional genomics and pharmacoepidemiology. Neuropharmacology. 2022;220:109262. doi:10.1016/j.neuropharm.2022.109262 36154842 PMC7618400

[8] Mazure CM, ed. Does Stress Cause Psychiatric Illness? American Psychiatric Press; 1995.

[9] Johansson L, Guo X, Hällström T, . Common psychosocial stressors in middle-aged women related to longstanding distress and increased risk of Alzheimer’s disease: a 38-year longitudinal population study. BMJ Open. 2013;3(9):e003142. doi:10.1136/bmjopen-2013-003142 24080094 PMC3787482

[10] Szczepanski SM, Knight RT. Insights into human behavior from lesions to the prefrontal cortex. Neuron. 2014;83(5):1002-1018. doi:10.1016/j.neuron.2014.08.011 25175878 PMC4156912

[11] Glantz LA, Lewis DA. Decreased dendritic spine density on prefrontal cortical pyramidal neurons in schizophrenia. Arch Gen Psychiatry. 2000;57(1):65-73. doi:10.1001/archpsyc.57.1.65 10632234

[12] Kolluri N, Sun Z, Sampson AR, Lewis DA. Lamina-specific reductions in dendritic spine density in the prefrontal cortex of subjects with schizophrenia. Am J Psychiatry. 2005;162(6):1200-1202. doi:10.1176/appi.ajp.162.6.1200 15930070

[13] Hof PR, Morrison JH. Neocortical neuronal subpopulations labeled by a monoclonal antibody to calbindin exhibit differential vulnerability in Alzheimer’s disease. Exp Neurol. 1991;111(3):293-301. doi:10.1016/0014-4886(91)90096-U 1999232

[14] Goldman-Rakic PS. Cellular basis of working memory. Neuron. 1995;14(3):477-485. doi:10.1016/0896-6273(95)90304-6 7695894

[15] Funahashi S, Bruce CJ, Goldman-Rakic PS. Mnemonic coding of visual space in the monkey’s dorsolateral prefrontal cortex. J Neurophysiol. 1989;61(2):331-349. doi:10.1152/jn.1989.61.2.331 2918358

[16] Wang M, Yang Y, Wang CJ, . NMDA receptors subserve working memory persistent neuronal firing In dorsolateral prefrontal cortex. Neuron. 2013;77(4):736-749. doi:10.1016/j.neuron.2012.12.032 23439125 PMC3584418

[17] Ferreira IL, Bajouco LM, Mota SI, Auberson YP, Oliveira CR, Rego AC. Amyloid beta peptide 1-42 disturbs intracellular calcium homeostasis through activation of GluN2B-containing N-methyl-d-aspartate receptors in cortical cultures. Cell Calcium. 2012;51(2):95-106. doi:10.1016/j.ceca.2011.11.008 22177709

[18] Ayalew M, Le-Niculescu H, Levey DF, . Convergent functional genomics of schizophrenia: from comprehensive understanding to genetic risk prediction. Mol Psychiatry. 2012;17(9):887-905. Epub ahead of print. doi:10.1038/mp.2012.37 22584867 PMC3427857

[19] Hohman TJ, Bush WS, Jiang L, ; Alzheimer’s Disease Genetics Consortium. Discovery of gene-gene interactions across multiple independent data sets of late onset Alzheimer disease from the Alzheimer Disease Genetics Consortium. Neurobiol Aging. 2016;38:141-150. doi:10.1016/j.neurobiolaging.2015.10.031 26827652 PMC4735733

[20] Pergola G, Di Carlo P, Andriola I, . Combined effect of genetic variants in the GluN2B coding gene (*GRIN2B*) on prefrontal function during working memory performance. Psychol Med. 2016;46(6):1135-1150. doi:10.1017/S0033291715002639 26690829

[21] Datta D, Leslie SN, Wang M, . Age-related calcium dysregulation linked with tau pathology and impaired cognition in non-human primates. Alzheimers Dement. 2021;17(6):920-932. doi:10.1002/alz.12325 33829643 PMC8195842

[22] Erraji-Benchekroun L, Underwood MD, Arango V, . Molecular aging in human prefrontal cortex is selective and continuous throughout adult life. Biol Psychiatry. 2005;57(5):549-558. doi:10.1016/j.biopsych.2004.10.034 15737671

[23] Reiken S, Sittenfeld L, Dridi H, Liu Y, Liu X, Marks AR. Alzheimer’s-like signaling in brains of COVID-19 patients. Alzheimers Dement. 2022;18(5):955-965. doi:10.1002/alz.12558 35112786 PMC9011576

[24] Alzheimer’s Association Calcium Hypothesis Workgroup. Calcium hypothesis of Alzheimer’s disease and brain aging: a framework for integrating new evidence into a comprehensive theory of pathogenesis. Alzheimers Dement. 2017;13(2):178-182.e17. doi:10.1016/j.jalz.2016.12.006 28061328

[25] Woo E, Sansing LH, Arnsten AFT, Datta D. Chronic stress weakens connectivity in the prefrontal cortex: architectural and molecular changes. Chronic Stress (Thousand Oaks). 2021;5:24705470211029254. doi:10.1177/24705470211029254 34485797 PMC8408896

[26] Catterall WA. Regulation of cardiac calcium channels in the fight-or-flight response. Curr Mol Pharmacol. 2015;8(1):12-21. doi:10.2174/1874467208666150507103417 25966697 PMC4664455

[27] Ling E, Nemesh J, Goldman M, . A concerted neuron-astrocyte program declines in ageing and schizophrenia. Nature. 2024;627(8004):604-611. doi:10.1002/cne.903590109 38448582 PMC10954558

[28] Maier SF, Amat J, Baratta MV, Paul E, Watkins LR. Behavioral control, the medial prefrontal cortex, and resilience. Dialogues Clin Neurosci. 2006;8(4):397-406. doi:10.31887/DCNS.2006.8.4/smaier 17290798 PMC3181837

[29] Datta D, Arnsten AFT. Loss of prefrontal cortical higher cognition with uncontrollable stress: molecular mechanisms, changes with age, and relevance to treatment. Brain Sci. 2019;9(5):113. doi:10.3390/brainsci9050113 31108855 PMC6562841

[30] Qin S, Hermans EJ, van Marle HJF, Lou J, Fernandez G. Acute psychological stress reduces working memory-related activity in the dorsolateral prefrontal cortex. Biol Psychiatry. 2009;66:25-32. doi:10.1016/j.biopsych.2009.03.006 19403118

[31] Dorow R, Horowski R, Paschelke G, Amin M. Severe anxiety induced by FG 7142, a beta-carboline ligand for benzodiazepine receptors. Lancet. 1983;2(8341):98-99. doi:10.1016/S0140-6736(83)90076-4 6134976

[32] Murphy BL, Arnsten AFT, Goldman-Rakic PS, Roth RH. Increased dopamine turnover in the prefrontal cortex impairs spatial working memory performance in rats and monkeys. Proc Natl Acad Sci U S A. 1996;93(3):1325-1329. doi:10.1073/pnas.93.3.1325 8577763 PMC40079

[33] Takamatsu H, Noda A, Kurumaji A, . A PET study following treatment with a pharmacological stressor, FG7142, in conscious rhesus monkeys. Brain Res. 2003;980(2):275-280. doi:10.1016/S0006-8993(03)02987-1 12867268

[34] Mikkelsen JD, Søderman A, Kiss A, Mirza N. Effects of benzodiazepines receptor agonists on the hypothalamic-pituitary-adrenocortical axis. Eur J Pharmacol. 2005;519(3):223-230. doi:10.1016/j.ejphar.2005.06.049 16125698

[35] Dazzi L, Vignone V, Seu E, Ladu S, Vacca G, Biggio G. Inhibition by venlafaxine of the increase in norepinephrine output in rat prefrontal cortex elicited by acute stress or by the anxiogenic drug FG 7142. J Psychopharmacol. 2002;16(2):125-131. doi:10.1177/026988110201600202 12095070

[36] Arion D, Enwright JF, Gonzalez-Burgos G, Lewis DA. Differential gene expression between callosal and ipsilateral projection neurons in the monkey dorsolateral prefrontal and posterior parietal cortices. Cereb Cortex. 2023;33(5):1581-1594. doi:10.1093/cercor/bhac157 35441221 PMC9977376

[37] Arion D, Corradi JP, Tang S, . Distinctive transcriptome alterations of prefrontal pyramidal neurons in schizophrenia and schizoaffective disorder. Mol Psychiatry. 2015;20(11):1397-1405. doi:10.1038/mp.2014.171 25560755 PMC4492919

[38] Dixon RE. Nanoscale organization, regulation, and dynamic reorganization of cardiac calcium channels. Front Physiol. 2022;12:810408. doi:10.3389/fphys.2021.810408 35069264 PMC8769284

[39] Wang M, Gamo NJ, Yang Y, . Neuronal basis of age-related working memory decline. Nature. 2011;476(7359):210-213. doi:10.1038/nature10243 21796118 PMC3193794

[40] Kim S, Yun HM, Baik JH, Chung KC, Nah SY, Rhim H. Functional interaction of neuronal Cav1.3 L-type calcium channel with ryanodine receptor type 2 in the rat hippocampus. J Biol Chem. 2007;282(45):32877-32889. doi:10.1074/jbc.M701418200 17823125

[41] Wang M, Ramos BP, Paspalas CD, . Alpha2A-adrenoceptors strengthen working memory networks by inhibiting cAMP-HCN channel signaling in prefrontal cortex. Cell. 2007;129(2):397-410. doi:10.1016/j.cell.2007.03.015 17448997

[42] Wu J, El-Hassar L, Datta D, . Interaction between HCN and slack channels regulates mPFC pyramidal cell excitability and working memory. bioRxiv. Posted online March 5, 2023. doi:10.1101/2023.03.04.529157 PMC13007569 37889366

[43] Brincat SL, Donoghue JA, Mahnke MK, Kornblith S, Lundqvist M, Miller EK. Interhemispheric transfer of working memories. Neuron. 2021;109(6):1055-1066.e4. doi:10.1016/j.neuron.2021.01.016 33561399 PMC9134350

[44] Shin MC, Nonaka K, Yamaga T, Wakita M, Akaike H, Akaike N. Calcium channel subtypes on glutamatergic mossy fiber terminals synapsing onto rat hippocampal CA3 neurons. J Neurophysiol. 2018;120(3):1264-1273. doi:10.1152/jn.00571.2017 29897859

[45] Bauer R, Timothy KW, Golden A. Update on the molecular genetics of timothy syndrome. Front Pediatr. 2021;9:668546. doi:10.3389/fped.2021.668546 34079780 PMC8165229

[46] Weitl N, Seifert R. Distinct interactions of human beta1- and beta2-adrenoceptors with isoproterenol, epinephrine, norepinephrine, and dopamine. J Pharmacol Exp Ther. 2008;327(3):760-769. doi:10.1124/jpet.108.143412 18772317

[47] Alexander JK, Hillier A, Smith RM, Tivarus ME, Beversdorf DQ. Beta-adrenergic modulation of cognitive flexibility during stress. J Cogn Neurosci. 2007;19(3):468-478. doi:10.1162/jocn.2007.19.3.468 17335395

[48] Breier A, Wolkowitz O, Pickar D. Stress and schizophrenia: Advances in neuropsychiatry and psychopharmacology. In: Tamminga C, Schult S, eds. Schizophrenia Research. Raven Press, Ltd; 1991.

[49] Katz MJ, Derby CA, Wang C, . Influence of perceived stress on incident amnestic mild cognitive impairment: results from the einstein aging study. Alzheimer Dis Assoc Disord. 2016;30(2):93-98. doi:10.1097/WAD.0000000000000125 26655068 PMC4877262

[50] Flatt JD, Gilsanz P, Quesenberry CPJ Jr, Albers KB, Whitmer RA. Post-traumatic stress disorder and risk of dementia among members of a health care delivery system. Alzheimers Dement. 2018;14(1):28-34. doi:10.1016/j.jalz.2017.04.014 28627380 PMC5729063

[51] Perlstein WM, Carter CS, Noll DC, Cohen JD. Relation of prefrontal cortex dysfunction to working memory and symptoms in schizophrenia. Am J Psychiatry. 2001;158(7):1105-1113. doi:10.1176/appi.ajp.158.7.1105 11431233

[52] Herold KG, Hussey JW, Dick IE. CACNA1C-related channelopathies. Handb Exp Pharmacol. 2023;279:159-181. doi:10.1007/164_2022_624 36598608 PMC10576998

[53] Yoshimizu T, Pan JQ, Mungenast AE, . Functional implications of a psychiatric risk variant within *CACNA1C* in induced human neurons. Mol Psychiatry. 2015;20(2):162-169. doi:10.1038/mp.2014.143 25403839 PMC4394050

[54] Song JHT, Lowe CB, Kingsley DM. Characterization of a human-specific tandem repeat associated with bipolar disorder and schizophrenia. Am J Hum Genet. 2018;103(3):421-430. doi:10.1016/j.ajhg.2018.07.011 30100087 PMC6128321

[55] Zink CF, Giegerich M, Prettyman GE, . Nimodipine improves cortical efficiency during working memory in healthy subjects. Transl Psychiatry. 2020;10(1):372. doi:10.1038/s41398-020-01066-z 33139710 PMC7606375

[56] Li D, He L. Association study between the NMDA receptor 2B subunit gene (*GRIN2B*) and schizophrenia: a HuGE review and meta-analysis. Genet Med. 2007;9(1):4-8. doi:10.1097/01.gim.0000250507.96760.4b 17224684

[57] Sabo SL, Lahr JM, Offer M, Weekes A, Sceniak MP. *GRIN2B*-related neurodevelopmental disorder: current understanding of pathophysiological mechanisms. Front Synaptic Neurosci. 2023;14:1090865. doi:10.3389/fnsyn.2022.1090865 36704660 PMC9873235

[58] Smedler E, Louhivuori L, Romanov RA, . Disrupted *Cacna1c* gene expression perturbs spontaneous Ca^2+^ activity causing abnormal brain development and increased anxiety. Proc Natl Acad Sci USA. 2022;119(7):e2108768119. doi:10.1073/pnas.2108768119 35135875 PMC8851547

[59] Krey JF, Paşca SP, Shcheglovitov A, . Timothy syndrome is associated with activity-dependent dendritic retraction in rodent and human neurons. Nat Neurosci. 2013;16(2):201-209. doi:10.1038/nn.3307 23313911 PMC3568452

[60] Birey F, Li MY, Gordon A, . Dissecting the molecular basis of human interneuron migration in forebrain assembloids from Timothy syndrome. Cell Stem Cell. 2022;29(2):248-264.e7. doi:10.1016/j.stem.2021.11.011 34990580 PMC13492730

[61] Baudic S, Barba GD, Thibaudet MC, Smagghe A, Remy P, Traykov L. Executive function deficits in early Alzheimer’s disease and their relations with episodic memory. Arch Clin Neuropsychol. 2006;21(1):15-21. doi:10.1016/j.acn.2005.07.002 16125364

[62] Murray PS, Kirkwood CM, Gray MC, . Hyperphosphorylated tau is elevated in Alzheimer’s disease with psychosis. J Alzheimers Dis. 2014;39(4):759-773. doi:10.3233/JAD-131166 24270207 PMC4034758

[63] Therriault J, Pascoal TA, Savard M, . Topographic distribution of amyloid-β, tau, and atrophy in patients with behavioral/dysexecutive Alzheimer disease. Neurology. 2021;96(1):e81-e92. doi:10.1212/WNL.0000000000011081 33093220 PMC7884976

[64] Leng K, Li E, Eser R, . Molecular characterization of selectively vulnerable neurons in Alzheimer’s disease. Nat Neurosci. 2021;24(2):276-287. doi:10.1038/s41593-020-00764-7 33432193 PMC7854528

[65] Bussière T, Giannakopoulos P, Bouras C, Perl DP, Morrison JH, Hof PR. Progressive degeneration of nonphosphorylated neurofilament protein-enriched pyramidal neurons predicts cognitive impairment in Alzheimer’s disease: stereologic analysis of prefrontal cortex area 9. J Comp Neurol. 2003;463(3):281-302. doi:10.1002/cne.10760 12820162

[66] Kritzer MF, Goldman-Rakic PS. Intrinsic circuit organization of the major layers and sublayers of the dorsolateral prefrontal cortex in the rhesus monkey. J Comp Neurol. 1995;359(1):131-143. doi:10.1002/cne.903590109 8557842

