# Supplementary material for: Key Roles of CACNA1C/Cav1.2 and CALB1/Calbindin in Prefrontal Neurons Altered in Cognitive Disorders
Source: JAMA Psychiatry. 2024 May 22;81(9):870–81. doi: 10.1001/jamapsychiatry.2024.1112 (PMC11112502; doi:10.1001/jamapsychiatry.2024.1112)
Supplement: Supplement 1. — eFigure 1. Schematic of methods used to perform single nucleus RNAseq from human dlPFC and a 3D portrayal of CALB1, CACNA1C and GRIN2B expression in the excitatory neurons of human and macaque dlPFC eFigure 2. Additional examples of multiple label immunofluorescence (MLIF) of Cav1.2, β1-AR, NMDAR-GluN2B, or SK3 and calbindin co-expression in macaque layer III dlPFC pyramidal cells eFigure 3. Additional examples of immunoEM ultrastructural localization of Cav1.2 channels, SK3 channels and β1-AR in layer III macaque dlPFC eFigure 4. Cav1.2 LTCCs and β1-ARs are expressed in dendritic spines near the SER spine apparatus in layer III macaque dlPFC eFigure 5. Cav1.2 LTCCs and β1-ARs are expressed in pyramidal cell-like dendritic shafts in layer III macaque dlPFC eFigure 6. SK3 channels are expressed on the plasma membrane of macaque layer III dlPFC spines and dendrites, and in astrocytes, but not in axons eFigure 7. Schematic of methods used for single unit recording from macaque dlPFC during a spatial working memory task, with iontophoretic delivery of drug to alter the local neurochemical environment eFigure 8. Estimate of drug concentration from iontophoresis at 20nA at 100 μm from electrode tip eFigure 9. Cav1.3 LTCCs have a more generalized expression pattern in macaque layer III dlPFC, including axonal labeling eFigure 10. Iontophoresis of the LTCC agonist, S-Bay K 6844, or the β1-AR agonist, xamoterol, markedly reduced delay-related firing and spatial tuning of dlPFC Delay cells eFigure 11. Iontophoresis of the LTCC antagonist, diltiazem, produced an inverted-U dose response, while the β1-AR antagonist, betaxolol, markedly enhanced delay-related firing and spatial tuning of dlPFC Delay cells eFigure 12. Blocking LTCCs prevents/reverses the reduction in firing from β1-AR stimulation eFigure 13. Iontophoresis of the SK channel blocker, NS8593, markedly enhanced the delay-related firing and spatial tuning of dlPFC Delay cells, while blockade of either SK or HCN channe [file jamapsychiatry-e241112-s001.pdf]

Supplemental Online Content

Datta D, Yang S, Joyce MKP, et al. Key roles of *CACNA1C*/Cav1.2 and *CALB1*/calbindin in prefrontal neurons altered in cognitive disorders. *JAMA Psychiatry*. Published online May 22, 2024. doi:10.1001/jamapsychiatry.2024.1112

eFigure 1. Schematic of methods used to perform single nucleus RNAseq from human dlPFC and a 3D portrayal of CALB1, CACNA1C and GRIN2B expression in the excitatory neurons of human and macaque dlPFC
eFigure 2. Additional examples of multiple label immunofluorescence (MLIF) of Cav1.2, β1-AR, NMDAR-GluN2B, or SK3 and calbindin co-expression in macaque layer III dlPFC pyramidal cells
eFigure 3. Additional examples of immunoEM ultrastructural localization of Cav1.2 channels, SK3 channels and □1-AR in layer III macaque dlPFC
eFigure 4. Cav1.2 LTCCs and β1-ARs are expressed in dendritic spines near the SER spine apparatus in layer III macaque dlPFC
eFigure 5. Cav1.2 LTCCs and β1-ARs are expressed in pyramidal cell-like dendritic shafts in layer III macaque dlPFC
eFigure 6. SK3 channels are expressed on the plasma membrane of macaque layer III dlPFC spines and dendrites, and in astrocytes, but not in axons
eFigure 7. Schematic of methods used for single unit recording from macaque dlPFC during a spatial working memory task, with iontophoretic delivery of drug to alter the local neurochemical environment
eFigure 8. Estimate of drug concentration from iontophoresis at 20nA at 100 □m from electrode tip
eFigure 9. Cav1.3 LTCCs have a more generalized expression pattern in macaque layer III dlPFC, including axonal labeling
eFigure 10. Iontophoresis of the LTCC agonist, S-Bay K 6844, or the β1-AR agonist, xamoterol, markedly reduced delay-related firing and spatial tuning of dlPFC Delay cells
eFigure 11. Iontophoresis of the LTCC antagonist, diltiazem, produced an inverted-U dose response, while the β1-AR antagonist, betaxolol, markedly enhanced delay-related firing and spatial tuning of dlPFC Delay cells
eFigure 12. Blocking LTCCs prevents/reverses the reduction in firing from β1-AR stimulation
eFigure 13. Iontophoresis of the SK channel blocker, NS8593, markedly enhanced the delay-related firing and spatial tuning of dlPFC Delay cells, while blockade of either SK or HCN channels blocked the effects of LTCC opening
eFigure 14. Behavioral data showing performance of the spatial delayed response test of spatial working memory under control vs. stress conditions, with pretreatment with vehicle or test drug
eFigure 15. RORB is widely expressed in dlPFC pyramidal cells, including in the CUX2A and CUX2C subgroups
eReferences

This supplemental material has been provided by the authors to give readers additional information about their work.

© 2024 Datta D et al. *JAMA Psychiatry.*

eMethods

All research was conducted according to USDA and NIH guidelines and approved by the Yale or University of Pittsburgh IACUC (for macaques) and the Partners Human Research Committee (for humans). For human dlPFC tissues, brain donors were recruited by the Harvard Brain Tissue Resource Center/NIH NeuroBioBank (HBTRC/NBB), in a community-based manner, across the USA. Human brain tissue was obtained from the HBTRC/NBB. The HBTRC procedures for informed consent by the donor's legal next-of-kin and distribution of de-identified post-mortem tissue samples and demographic and clinical data for research purposes are approved by the Mass General Brigham Institutional Review Board. Post-mortem tissue collection followed the provisions of the United States Uniform Anatomical Gift Act of 2006 described in the California Health and Safety Code section 7150 and other applicable state and federal laws and regulations. Federal regulation 45 CFR 46 and associated guidance indicates that the generation of data from de-identified post-mortem specimens does not constitute human participant research that requires institutional review board review.

Transcriptomics

The methods for transcriptomic analyses are summarized in graphic form in eFigure 1, below. Fresh-frozen postmortem human dlPFC tissue from 50 neurotypical donors from the Harvard Brain Tissue Resource Center/NIH NeuroBioBank was processed for single nucleus RNA-seq (10x 3' v3). These brains had been previously selected as matched controls for a separate study on brain differences in schizophrenia; this sample had the following demographics: 22% female, 78% male; ages 50-88 years, all Caucasian with the exception of one American Indian/Alaskan Native and one Asian/Pacific Islander. The generation of expression profiles will be described in detail in a forthcoming manuscript from Ling, Goldman, Berretta and McCarroll. In brief, we developed and optimized a workflow for creating and analyzing "brain nuclei villages" – nuclei sampled from the dlPFC (BA46) from 20 different donors per village. Fresh-frozen postmortem macaque dlPFC tissue was provided by the Lewis group at the University of Pittsburgh and provided to the Krienen lab at Princeton. Fresh-frozen brain tissue (dlPFC containing both banks of the principal sulcus area 46) was obtained from two experimentally-naïve, female, adult (6.6 and 8.2 years of age) Rhesus macaque monkeys. Animals were deeply anesthetized and perfused with artificial cerebral spinal as previously described[1] in accordance with USDA and NIH guidelines and with the approval of the University of Pittsburgh IACUC.

dlPFC tissue was dissected from each donor, obtaining a similar mass of tissue from each specimen while being careful to represent all cortical layers. The frozen tissue samples are then immediately pooled for simultaneous isolation of their nuclei; all subsequent processing steps – including nuclear isolation, encapsulation in droplets, and preparation of snRNA-seq libraries – involve all of the donors together. This "Dropulation" workflow allows us to minimize experimental variability, including any technical effects on mRNA ascertainment and any effects of cell-free ambient RNA. Each nucleus in these experiments was then re-assigned to its donor-of-origin using combinations of hundreds of transcribed SNPs; though the individual SNP alleles are shared among many donors, the combinations of many SNPs are unique to each donor in the cohort.

Nuclei with fewer than 500 genes or 1000 transcripts were removed from the analysis. Independent component analysis (ICA, using the fastICA package in R) was performed on the

excitatory neurons, using the filtered digital gene expression matrix (DGE) after normalization and variable gene selection following procedures described in Saunders et al. 2018[2]. Louvain clustering (resolution 0.1, nearest neighbors = 25) was performed using the top 60 ICs. This resulted in 20 clusters, including 5 major GABAergic clusters (identified by expression of *GAD1* or *GAD2*, and 7 major glutamatergic (*SLC17A7+*) clusters, as well as astrocytes, endothelial cells, microglia, oligodendrocytes and polydendrocytes.

ICA and Louvain clustering was repeated on each of the 7 glutamatergic clusters, resulting in further subdivisions of the major glutamatergic types for a total of 12 subclusters. Cell and gene weights on independent components (ICs) were manually inspected for evidence of cell-cell doublets or non-excitatory neurons, both of which were removed from the DGE. ICs that reflected artifactual signals or batch effects were identified and excluded, and Louvain clustering was performed on the retained ICs. Within each cluster, transcripts for each gene were summed across cells of that cluster. The resulting "metacell" counts were normalized to the total number of transcripts, then scaled to counts per 100,000 and log10 transformed. All data from the human cohort will be deposited in NeMO under accession number nemo:dat-bmx7s1t in a forthcoming manuscript from Ling *et al.*

Multiple label immunofluorescence-

Immunofluorescence staining was carried out on free-floating sections in the same rhesus macaques used for immunoEM, or in tissue from two younger macaques (female, 8 and 10 years), an age when CB labeling in pyramidal neurons is more robust[3]. The sections were washed in 1X TBS (pH 7.4, 4 x 15 min) for 1 h at RT. Sections with GluN2B or SK3 labeling underwent antigen retrieval in sodium citrate (10mM, JT Baker cat # 3646-01) using a water bath at 70-80°C (30min), followed by washes and incubation for 1h in 50mM glycine (Sigma, cat # G-7126, in 0.1M PB). If streptavidin-amplified, sections were then blocked with Component A and Component B for 20 min each using Invitrogen's Endogenous Biotin Blocking kit, and then blocked for 1 h at RT in 1X TBS containing 5% bovine serum albumin, 2% Triton X-100, and 10% species-specific normal serum. Sections were incubated for 48 h at 4°C with specific primary antibodies: $Cav_{1.2}$ (rabbit anti-$Cav_{1.2}$ (CACNA1C) at 1:100 [cat# ACC-003, RRID:AB_2039771, Alomone]), β1-AR (goat anti-beta 1 adrenergic receptor at 1:100 [cat# ab77189, RRID:AB_1523202, Abcam]), Calbindin (mouse anti-calbindin D-28k at 1:100 [cat# 300, RRID:AB_10000347, Swant Antibodies]), SK3 (rabbit anti-KCNN3, $K_{Ca}2.3$ at 1:100 [cat# APC-025, RRID:AB_2040130, Alomone]), or GluN2B at 1:100 (rabbit anti-NMDAR2B [cat# AGC-003, RID:AB_2040028, Alomone] in dilution buffer (2% bovine serum albumin, 2% Triton X-100, and 1% species-specific normal serum). Appropriate secondary antibodies in dilution buffer were used at 1:100 or 1:1000, overnight at 4°C (donkey anti-mouse AF546, Invitrogen cat#A10036; donkey anti-goat AF594, Invitrogen cat#A11058; donkey anti-goat AF488, Invitrogen, cat#A11055; donkey anti-rabbit IgG antibody biotinylated, FisherSci, cat#16027; donkey anti-goat IgG antibody biotinylated, FisherSci, cat#A16003; goat anti-mouse AF647, Invitrogen, cat# A32728; goat anti-rabbit AF488, Invitrogen, cat# A11008). The $Cav_{1.2}$, SK3 and $Cav_{1.3}$ antibodies have all been validated in conditional knockout animals[4-6]. All subsequent steps were performed in the dark and at RT. The sections were first washed in 1X TBS (pH 7.4, 4 x 15 minutes), followed by incubation in Streptavidin, AF488 conjugate (0.5μg/ml, ThermoFisher, Cat#S11225) for 1 h, if using streptavidin amplification. The sections were washed in 1X TBS (pH 7.4, 3 x 5 minutes) and some sections were counterstained with Hoechst 33342 for 10 minutes (1:10,000,

ThermoFisher, Cat# H3570). The sections were washed in 1X TBS (pH 7.4, 3 x 10 minutes) before mounting onto slides using ProLong Gold Antifade Mountant (Invitrogen, Cat# P36930).

Confocal images were acquired using a Leica TCS SP8 Gated STED 3X super resolution microscope, with the HC PL APO 100X/1.40 oil white objective (Leica) and HCX PL APO CS 63X/1.40 oil white objective (Leica), or a Zeiss LSM 880 Airyscan with the Plan-Apochromat 20x/0.8 M27 objective. Z-stacks were obtained with ~0.3-μm steps under laser excitation at 407nm, 488nm, 543nm, 561nm, or 633nm, as determined by the antibody combination being imaged. Emission filter bandwidths and sequential scanning acquisition were set up in order to avoid possible spectral overlap between fluorophores. Confocal images were deconvoluted with Hugyens Professional version 22.04 (Scientific Volume Imaging, The Netherlands, http://svi.nl) using the theoretical PSF. The confocal Z-stacks were processed into maximum intensity Z-projections using Fiji and background subtraction with a rolling ball radius of 50 pixels and 100 pixels was applied to all 63X and 100X channels, respectively (applied to the entire panel). Images were adjusted for brightness and contrast, labeled, and assembled into a figure using Adobe Photoshop CS5 Extended (version 12.0.4 ×64, Adobe Systems Incorporated) and Adobe Illustrator 25.0.1 (Adobe Systems Incorporated version 27.7).

To quantify the percent of CB cells that were positive for $Cav_{1.2}$, β1-AR, NMDAR-GluN2B, or SK3, we sampled multiple fields of view in layer III of each section. We used the isolated CB channel to trace lightly labeled CB+ neurons with characteristic pyramidal-like morphology, and measured the mean grey value of these traces in the isolated channel containing the second protein (e.g., NMDAR-GluN2B). We compared the mean grey value in each CB pyramidal neuron trace to the average mean grey value sampled from the neuropil. CB pyramidal cells were deemed colocalized with the second protein if the mean grey value exceeded the average from the sampled neuropil.

Single label IHC-

The expression pattern of the transcription factor, RORB, in macaque dlPFC was examined due to its correlation with Alzheimer's pathology (see eFig. 11 below). This analysis utilized immunoperoxidase immunohistochemistry, where sections of macaque dlPFC (26-31y) were transferred for 1 hr to Tris-buffered saline (TBS) containing 5% bovine serum albumin, plus 0.05% Triton X-100 to block non-specific reactivity, and incubated in primary antibody for RORB (1:250 dilution; rabbit, HPA008393, Millipore Sigma) in TBS for 48 hr at 4°C. The tissue sections were incubated in goat anti-rabbit biotinylated antibody (Vector Laboratories) at 1:300 in TBS for 2 hr, and developed using the Elite ABC kit (Vector Laboratories) and diaminobenzidine (DAB) as a chromogen.

ImmunoEM-

We used the pre-embedding immunoperoxidase immunoEM approach given its high sensitivity and thus suitability for quantitative studies. As calbindin is a cytosolic protein, labeling would fill the entire cell, and thus immunoEM of calbindin was not performed. The research utilized dlPFC perfusion-fixed tissue from two rhesus macaques (female, ages 18 and 19yrs). Previously characterized primary antibodies raised in rabbit or mice were used, and complexed with species-specific goat secondary antibodies. The following primary antibodies were used: 1) rabbit anti-$Cav_{1.2}$ (CACNA1C) at 1:200 (cat# ACC-003, RRID:AB_2039771, Alomone); 2) rabbit

© 2024 Datta D et al. *JAMA Psychiatry.*

anti-β1-adrenergic receptor at 1:100 (cat # AAR-023, RRID:AB_2340886, Alomone); 3) rabbit anti-SK3 (KCNN3, $K_{Ca}2.3$) at 1:100 (cat # APC-025, RRID:AB_2040130, Alomone); 4) rabbit anti-$Cav_{1.3}$ (CACNA1D) at 1:100 (cat # ACC-005, RRID:AB_2039775, Alomone). The specificity and selectivity of the primary antibodies have been characterized using immunohistochemistry and immunofluorescence approaches in various tissue types[7-11].

Unlike the other antibodies, the β1-AR antibody (rabbit anti-β1 adrenergic receptor at 1:100 [cat#AAR023, RRID:AB_2340886, Alomone]) was not knockout-validated, and thus we performed an additional control, utilizing the manufacturer's bespoke blocking peptide (Alomone, cat # BLP-AR023). Tissue sections were processed for β1-AR immunofluorescence, following the antigen retrieval and blocking steps outlined above, and test sections were incubated with the primary antibody for β1-AR and the blocking peptide (at 10x the concentration of the primary antibody, as instructed). The sections were then paired with goat anti-rabbit AF488 (Invitrogen). As shown here, neuronal labeling was evident when the blocking peptide was omitted (A); negligible labeling was observed in the test section that had been processed with the blocking peptide (B).

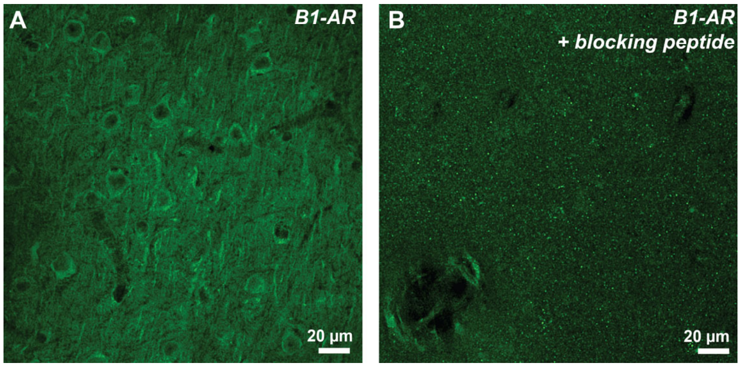

In contrast to the β1-AR antibody, the $Cav_{1.2}$, SK3 and $Cav_{1.3}$ antibodies have all been validated in conditional knockout animals[4-6]. Normal sera and IgG-free BSA were purchased from Jackson Immunoresearch. All chemicals and supplies for electron microscopy were purchased from Sigma Aldrich and Electron Microscopy Sciences, respectively.

Sections were incubated for 72 h at 4 °C with primary antibodies in TBS, and transferred for 2 h at room temperature to species-specific biotinylated Fab' or $F(ab')_2$ fragments in TBS. In order to reveal immunoperoxidase labeling, sections were incubated with the avidin-biotin peroxidase complex (ABC) (1:300; Vector Laboratories, Burlingame, CA, United States of America) and then visualized in 0.025% Ni-intensified 3,3-diaminobenzidine tetrahydrochloride (DAB; Sigma Aldrich, St. Louis, MO, United States of America) as a chromogen in 100mM PB with the addition of 0.005% hydrogen peroxide for 6-12 minutes, depending on the target of interest. After the DAB reaction, sections were exposed to osmification (concentration 1%), dehydration through a series of increasing ethanol concentrations (70-100%), and infiltrated with propylene oxide. Tissue sections were counterstained with 1% uranyl acetate in 70% ethanol. Standard epoxy resin embedding followed typical immunoEM procedures followed by polymerization at 60°C for 48 h. Omission of primary antibodies or substitution with non-immune serum resulted in complete lack of immunoperoxidase labeling. Similarly, labeling was nullified when blocking the biotinylated probes with avidin/biotin. Blocks containing dlPFC layer III were sampled and mounted onto resin blocks. The specimens were cut into 50 nm sections using an ultramicrotome (Leica) and analyzed under a Talos L120C transmission electron microscope (Thermo Fisher Scientific). Several plastic blocks of each brain were examined using the 4th to 12th surface-most sections of each block (i.e., 200-600 nm), in order to sample the superficial component of sections, avoiding penetration artifacts. Structures were digitally captured at x25,000-x100,000 magnification with a Ceta CMOS camera and individual panels were adjusted

© 2024 Datta D et al. *JAMA Psychiatry.*

for brightness and contrast using Adobe Photoshop and Illustrator CC.2020.01 image editing software (Adobe Systems Inc.). Approximately, 2000 micrographs of selected areas of neuropil with immunopositive profiles were used for analyses with well-defined criteria.

For quantification of the distance from the SER to membrane-bound $Cav_{1.2}$, SK3, or β1-AR, we selected photomicrographs of spines with synaptic or extrasynaptic membrane-associated immunoperoxidase label and a discernable spine apparatus. Using Reconstruct[12], we calibrated the pixel size of each image and measured the shortest distance from the center of the membrane bearing the immunoperoxidase label to the spine apparatus. Data were analyzed and plotted using Prism 9 (Graphpad Software, San Diego CA). Means are reported with standard error of the mean.

The criteria summarized in Peters et al. (A. Peters, S. L. Palay, H. D. Webster, *The Fine Structure of the Nervous System: Neurons and Their Supporting Cells*. Oxford Univ. Press, New York, NY, 1991) were utilized for profile identification. Dendritic spines in the PFC are typically long and thin, devoid of mitochondria, with the presence of a noticeable postsynaptic density at asymmetric synapses, and often containing an elaborated spine apparatus, the extension of the smooth endoplasmic reticulum into the spine. Dendritic shafts were typically round in perpendicular planes or irregular shaped when assessed in horizontal planes, usually containing mitochondria and numerous tubular and pleomorphic cellular organelles. Depending on the proximity to axon terminals, various dendritic shafts received synaptic inputs. Axon terminals contained accumulations of synaptic vesicles and the axoplasm of these terminals usually contained neurofilaments and mitochondria. The synaptic innervations made by these axon terminals were either asymmetric, containing spherical vesicles, or symmetric, containing pleomorphic vesicles, with typical differences in postsynaptic density, respectively. Unmyelinated axons were small, round processes with a predominantly even and regular shape, traversing the neuropil in a straight orientation, often forming bundles in perpendicular planes. Astrocytic processes were typically of irregular morphology, forming contours that filled the empty space around neuronal elements.

Physiology-

The methods for single unit recordings from the cognitively-engaged macaque dlPFC, coupled with iontophoresis of drug locally onto the neuron being recorded, are summarized in eFigure 7. Two rhesus monkeys (*Macaca mulatta;* monkey T, 24 years old, female; monkey C, 15 years old, male) were trained on the ODR task (Fig. 4A), where the monkey fixates on a central point for 0.5sec to initiate a trial; a cue then comes on for 0.5sec in one of eight locations, randomly selected by the computer. The monkey must remember the spatial location over a delay period of 2.5sec while maintaining fixation centrally. At the end of the delay, the fixation point is extinguished, and the monkey can move its eyes to the remembered location within 1sec for juice reward. The monkey can then fixate on the central spot to initiate a new trial. Thus, the contents of working memory must be constantly updated on each trial. Iontophoretic, single unit recordings were made from the dlPFC subregion near the caudal principal sulcus, anterior to the frontal eye fields. This subregion of the dlPFC is known to be essential for the accurate performance of this task, as temporary lesions to this area markedly impair performance[13-16]. The iontophoretic electrode contains a central carbon fiber for neuronal recording, surrounded by 7 glass micropipettes for ejection of drug solutions using minute amounts of electric current (+10-60nA). Note that drugs must contain an electric charge to be amenable to this technique.

© 2024 Datta D et al. *JAMA Psychiatry.*

Each compound was dissolved at 0.01 M concentration in either sterile water or DMSO. Neural signals were digitized (15kHz; micro 1401, Cambridge Electronics Design, Cambridge, UK) and acquired using the Spike2 software (CED, Cambridge, UK).

Only Delay cells were selected for subsequent drug testing. Delay cells show persistent firing across the delay period if a cue had been presented at its "preferred direction", e.g. 180º, but not other, "nonpreferred" directions. This spatially tuned, persistent firing generates a mental representation of visual space that is foundational to visuospatial working memory[14]. For the sake of space, figures only show the neuronal firing for a neuron's preferred direction and just one nonpreferred direction in the opposing direction. The spatial tuning of a Delay cell was analyzed using a d' measure of spatial selectivity during the delay epoch [$d' = mean_{pref} - mean_{nonpref} / \sqrt{(sd^2 pref + sd^2_{nonpref})}$]. Delay cells often fire to the cue and to the response as well as across the delay epoch, and their persistent firing is coincident with gamma bursts in local field potentials (LFPs) that average the electrical activity of a larger expanse of neurons[17,18]. However, as iontophoresis ejects a very small amount of drug in a very limited area, LFPs are usually only altered by very large doses of lipophilic compounds. Please note that there are other types of task-related neurons in the primate dlPFC, e.g. Fixation, Cue, Response and Response Feedback cells, but these have very different molecular dependencies than Delay cells, e.g. relying more on AMPAR than NMDAR-GluN2B neurotransmission. Given the time-intensive nature of this research, the current study focused on Delay cells due to their importance to working memory[13], their localization in dlPFC layer III[13] and their known dependency on NMDAR-GluN2B (*GRIN2B*) neurotransmission[19]. Statistical analyses utilized repeated measures ANOVA with two-tailed significance.

Cognitive behavior-

Rhesus monkeys (n=6, 10-22 years, 5 male, 1 female) were pretrained on the delayed response task, a test of visuospatial working memory performed with a manual response in a Wisconsin General Test Apparatus. Monkeys test for highly palatable food rewards, limiting the need for food regulation. This version of the task allows free movement that is more appropriate for studies of the effects of mild stress on cognitive performance. Monkeys were tested by an experimenter who was highly familiar with the normative behavior of each animal, but unaware of drug treatment conditions. Animals were rated for changes in sedation/agitation and aggression using 9 point rating scales.

For each monkey, a dose of the anxiogenic pharmacological stressor, FG7142 (0.5-1.0 mg/kg, i.m. 30 min. before testing), was found that reliably impaired accuracy of performance, but was mild enough that the monkey would work for the full 30 trials that made up a daily test session. Please note that FG7142 creates a classic stress response in humans[20], monkeys [21,22] and rodents[23], including cortisol/corticosterone release[20,22,24] and increased NE release in the PFC e.g.[25], and discriminates similar to other stressors[23]. However, unlike many environmental stressors, the dose can be titrated to find a humane paradigm, where the amount that impairs accuracy, but retains motivation to perform the task for all 30 trials, and allows direct comparison to previous research using this compound. A dose of the β1-AR antagonist, betaxolol (between 0.001-0.1 mg/kg, p.o., one hour before testing) and of nimodipine (0.1-1.0 mg/kg, p.o., one hour before testing) was identified for each animal that did not improve performance on its own, in order that subsequent challenge of the stress response could not be explained by simple additive effects of the two treatments. Following these initial dose-finding experiments, the effects of

systemic pretreatment (p.o., one hour before testing) with the β1-AR antagonist, betaxolol, or the LTCC blocker nimodipine were used to challenge the effects of FG7142 or vehicle (veh) i.m. 30 minutes before testing on spatial working memory performance. Statistical analyses employed repeated-measures 2-ANOVA-R with Tukey's multiple comparisons test for post-hoc comparisons with two-tailed significance.

© 2024 Datta D et al. *JAMA Psychiatry.*

eFigure 1

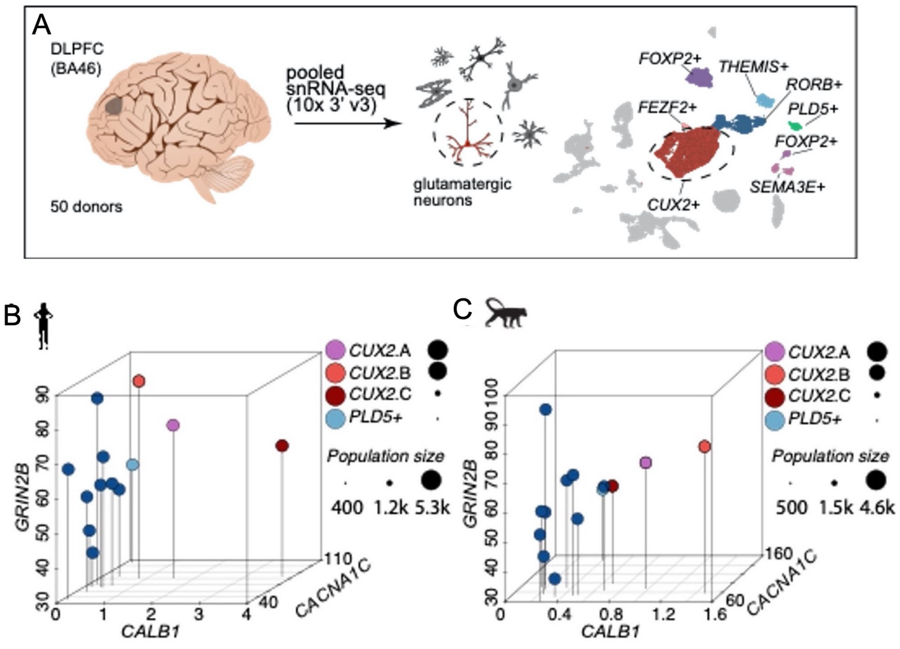

eFigure 1- Schematic of methods used to perform single nucleus RNAseq from human dlPFC and a 3D portrayal of *CALB1, CACNA1C* and *GRIN2B* expression in the excitatory neurons of human and macaque dlPFC. A. Excitatory neurons were identified by expression of the canonical markers *SLC17A7* and *VGLUT1*. **B.** Transcriptomic analyses of human dlPFC pyramidal cells, showing expression levels of *CALB1* (encoding the calcium binding protein, calbindin), *CACNA1C* (encoding the LTCC Cav$_{1.2}$), and *GRIN2B* (encoding the NMDA receptors with a GluN2B subunits that flux high levels of calcium). The superficial layer *CUX2*-expressing cell groups are shown in red, pink and purple; the PLD5 expressing cells in light blue; other excitatory neuron subgroups in dark blue. Population sizes are represented in the key, expressed as number of nuclei out of 20,000. The *CUX2*-expressing cells have higher *CALB1* than other excitatory cells, consistent with their expressing high levels of calcium-related signaling proteins, where *CACNA1C* (Cav$_{1.2}$) and *GRIN2B* (NMDAR-GluN2B) would flux high levels of calcium into the neuron. As shown in the main text, this would reduce neuronal firing when calcium opens SK potassium channels, which are also highly expressed in these neurons (*KCNN3*). **C.** Same as (B) but in macaque dlPFC. Note that there is a very group of excitatory cells in both species, highlit in light blue, that express the highest levels of *CACNA1C* that express *PLD5* but not *CUX2,* and have less *CALB1*.

© 2024 Datta D et al. *JAMA Psychiatry.*

eFigure 2

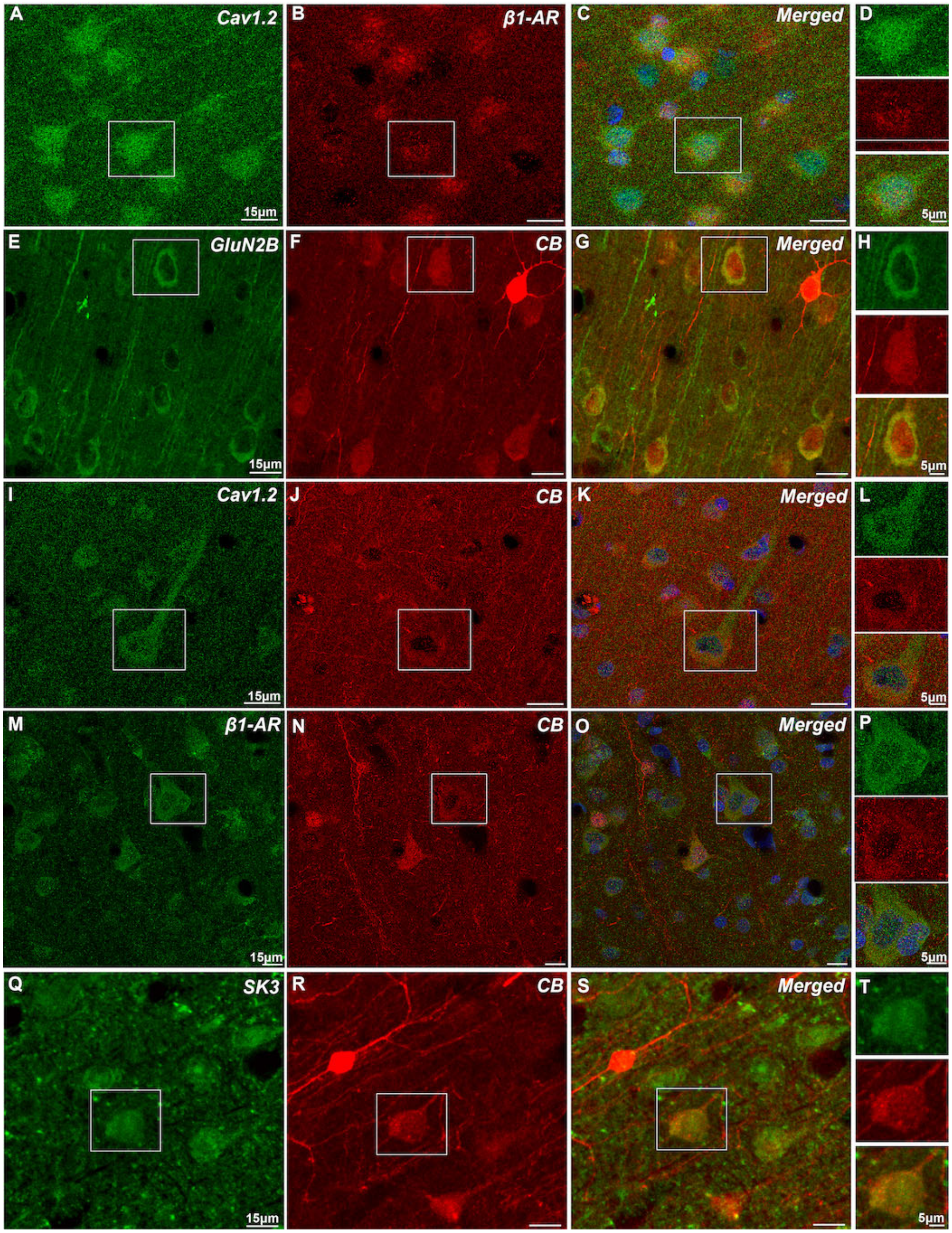

eFigure 2- Additional examples of multiple label immunofluorescence (MLIF) of $Cav_{1.2}$, β1-AR, NMDAR-GluN2B, or SK3 and calbindin co-expression in macaque layer III dlPFC pyramidal cells. **(A-D)** MLIF shows co-expression of $Cav_{1.2}$ and β1-AR in layer III pyramidal cells in macaque dlPFC; biotin-streptavidin was used to amplify the $Cav_{1.2}$ signal. **(E-H)** MLIF shows co-expression of NMDAR-GluN2B and calbindin (CB) in layer III pyramidal cells in macaque dlPFC. **(I-L)** MLIF shows co-expression of $Cav_{1.2}$ and calbindin in layer III pyramidal cells in macaque dlPFC; biotin-streptavidin was used to amplify the $Cav_{1.2}$ signal. **(M-P)** MLIF

© 2024 Datta D et al. *JAMA Psychiatry.*

shows co-expression of β1-AR and calbindin in layer III pyramidal cells in macaque dlPFC; biotin-streptavidin was used to amplify the β1-AR signal. Note that *ADRB2* was undetectable in *CALB1*-expressing pyramidal cells and thus this receptor was not examined here. **(Q-T)** MLIF shows co-expression of SK3 and calbindin in layer III pyramidal cells in macaque dlPFC. Note that calbindin expression is much higher in inhibitory interneurons than pyramidal cells, (e.g. F, R). Where present, blue labeling is the Hoechst nuclear counter stain.

© 2024 Datta D et al. *JAMA Psychiatry.*

eFigure 3

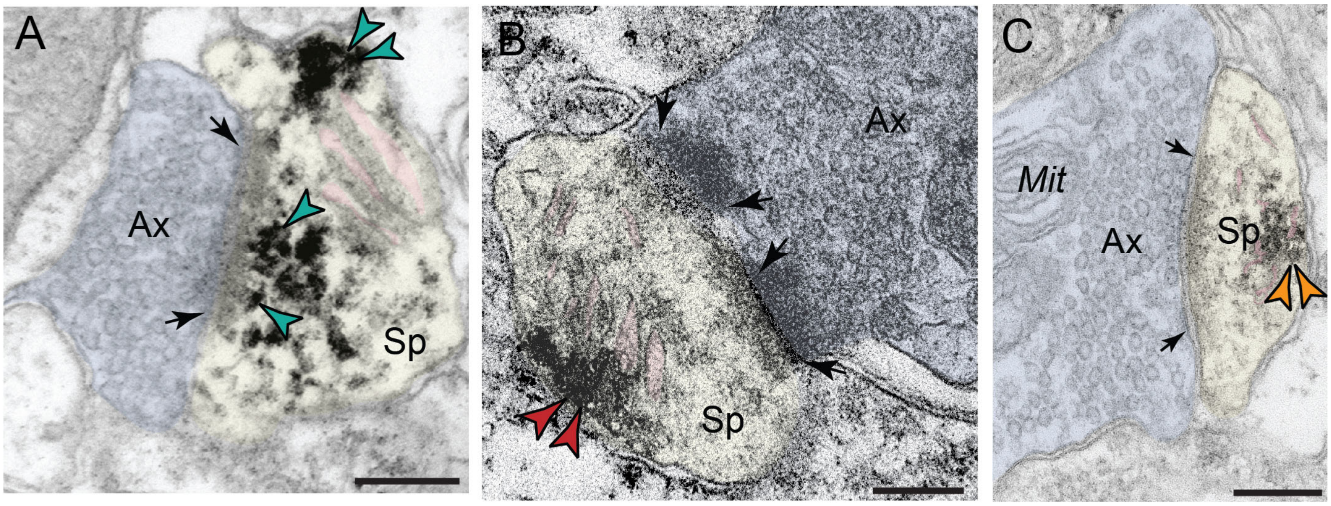

eFig. 3- Additional examples of immunoEM ultrastructural localization of Cav1.2 channels, SK3 channels and β1-AR in layer III macaque dlPFC. Cav1.2 (**A, teal arrowheads**), β1-AR (**B, red arrowheads**) and SK3 channels (**C, orange arrowheads**) can all be seen in dendritic spines receiving asymmetric (presumed glutamatergic) synapses, often localized on the plasma membrane near the calcium-storing/releasing smooth endoplasmic reticulum (SER; termed the spine apparatus when it is elaborated in the dendritic spine). The SER spine apparatus is highlit with pink pseudocoloring. Note that the Cav1.2 labeling can be seen near the post-synaptic density (PSD) and near the SER. Spines (Sp) are pseudocolored yellow; axon terminals (Ax) are pseudocolored blue; Mit=mitochondrion. Scale bars: 200nm.

© 2024 Datta D et al. *JAMA Psychiatry.*

eFigure 4

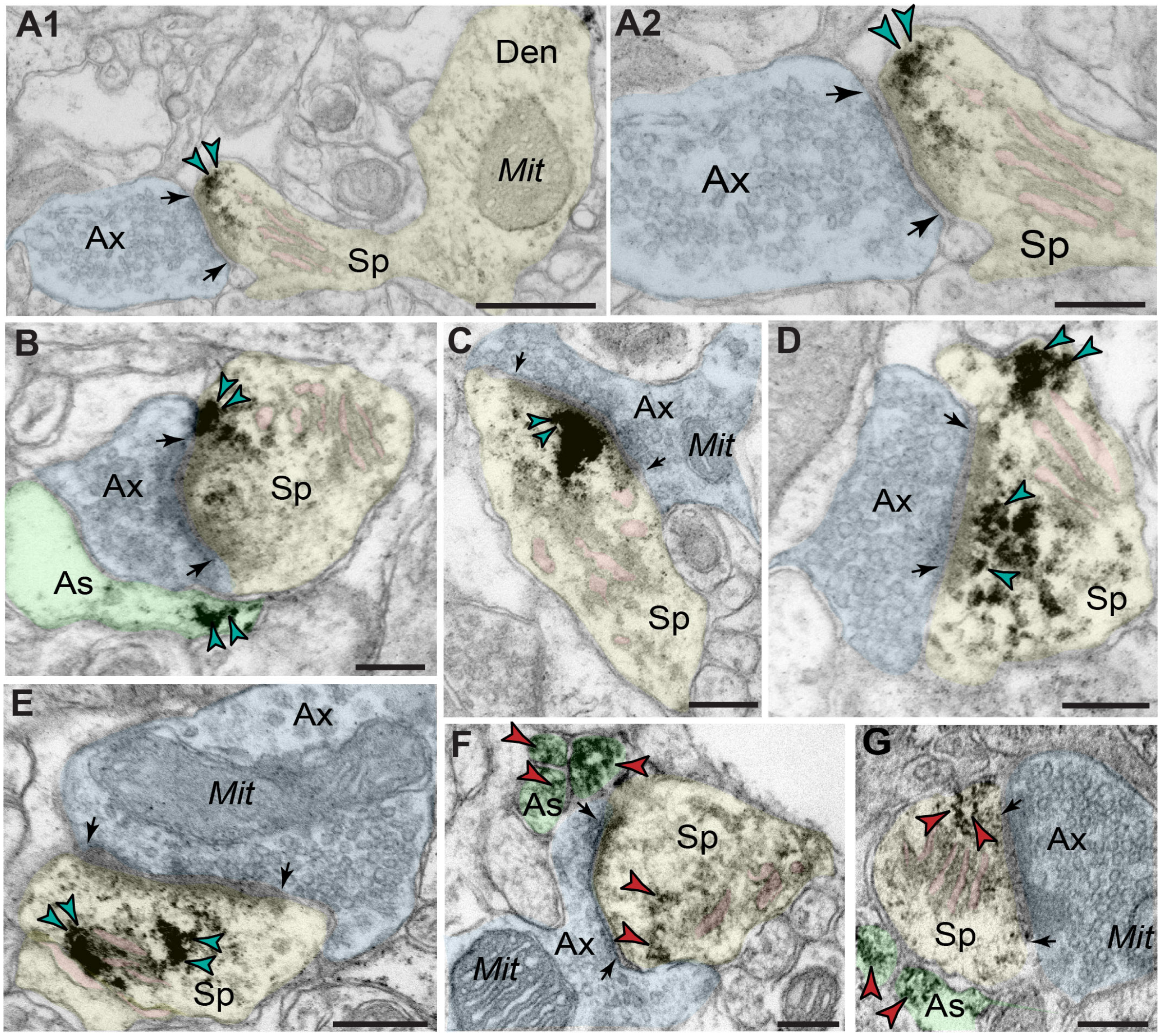

eFig. 4- Cav1.2 LTCCs and β1-ARs are expressed in dendritic spines near the SER spine apparatus in layer III macaque dlPFC. (A-E) $Cav_{1.2}$ immunolabeling (indicated by teal arrowheads) is prominently expressed in dendritic spines (pseudocolored yellow), frequently near the calcium-storing smooth endoplasmic reticulum (SER) spine apparatus (pseudocolored pink). $Cav_{1.2}$ are seen on the plasma membrane at perisynaptic locations **(A, B, D)**, and flanking the excitatory synapse, as well as within the post-synaptic density **(C, D)**. In **A1**, a dendritic spine emanaties from a dendritic shaft of a pyramidal cell in dlPFC layer III neuropil, with $Cav_{1.2}$ immunolabeling observed perisynaptically near the glutamatergic-like synapse (higher magnification image of same dendritic spine profile in **A2**). $Cav_{1.2}$ immunolabeling can also be seen amongst the membranes of the SER spine apparatus **(E)**. Astrocytic labeling (pseudocolored in green) for $Cav_{1.2}$ can also be seen in **B**. **(F-G)** Additional examples of β1-AR immunolabeling (indicated by red arrowheads) on the plasma membrane of a layer III dendritic spine, near the synapse and the SER spine apparatus. β1-AR immunolabeling is also observed on the astrocytic glial leaflets (pseudocolored in green) near the dendritic spine. All dendritic spines receive axospinous Type I asymmetric glutamatergic-like synapses, which are between arrows. As, astrocyte; Ax, axon; Mit, mitochondria; Sp, dendritic spine; Den, dendrite. Scale bars, 200 nm.

© 2024 Datta D et al. *JAMA Psychiatry.*

eFigure 5

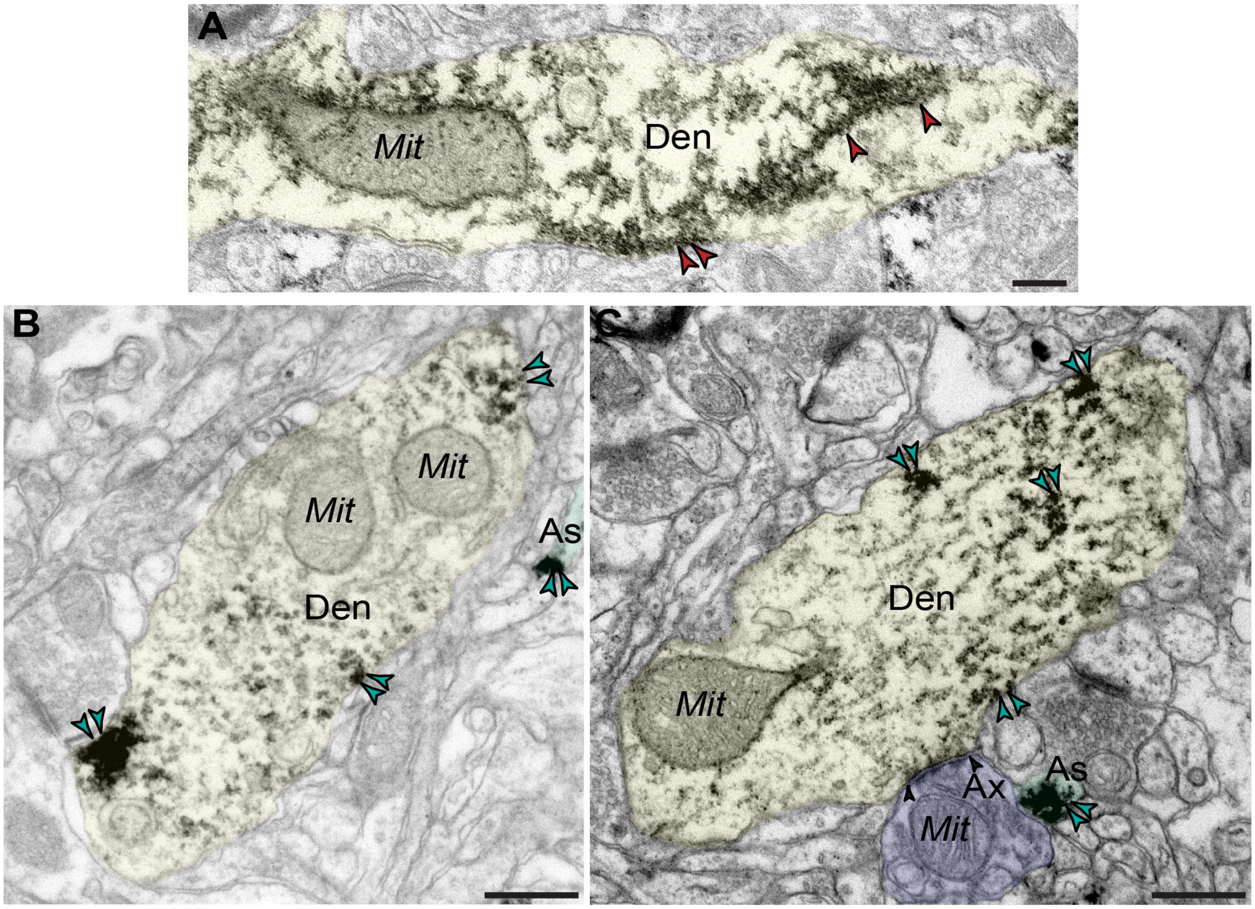

eFig. 5- Cav1.2 LTCCs and β1-ARs are expressed in pyramidal cell-like dendritic shafts in layer III macaque dlPFC. (A) An example of β1-AR immunolabeling (red arrowheads) on the plasma membrane of a pyramidal-like dendrite (pseudocolored yellow). The β1-AR labeling also appears to cluster along microtubules, suggestive of receptor trafficking within the dendrite. **(B-C)** Cav1.2 immunolabeling (teal arrowheads) can also be found on the plasma membrane of dendritic shafts with a pyramidal cell-like morphology, as well as on microtubules within the dendrite. In **B**, the dendrite is cross-sectioned, and the labeling on microtubules thus appear as puncta, while **C** shows a longitudinal view of a dendrite, where the labeling on microtubules can be seen in parallel bundles, likely undergoing intracellular trafficking. In **C**, the pyramidal cell dendrite receives a symmetric Type II synapse from a putative GABAergic interneuron (pseudocolored purple), consistent with pyramidal cell dendrites receiving inhibitory synapses. Labeling of astrocytic processes can also be seen **(B,C)**. In contrast to dendrites and spines, there was little or no labeling of axons. As, astrocyte; Ax, axon; Mit, mitochondria; Den, dendrite. Scale bars, 200nm.

© 2024 Datta D et al. *JAMA Psychiatry.*

eFigure 6

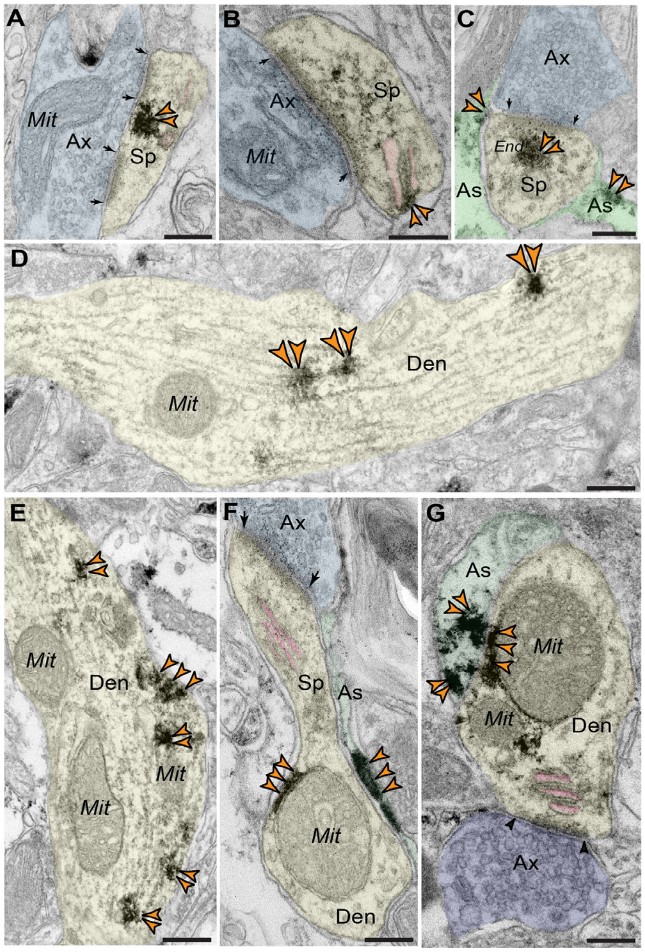

eFig. 6- SK3 channels are expressed on the plasma membrane of macaque layer III dlPFC spines and dendrites, and in astrocytes, but not in axons. SK3 channels (orange arrowheads) are expressed on the plasma membrane of spines and dendrites (pseudocolored yellow), and in astrocytes (pseudocolored green), but not in axons. SK3 immunolabeling is seen in spines subjacent to the asymmetric post-synaptic density **(A, C)**, and in extrasynaptic locations **(B)**, often near the calcium-storing spine apparatus **(A, B,** pseudocolored pink**)**. Although it is not technically feasible to test whether Cav1.2induced calcium signaling in dlPFC spines drives internal calcium release from the SER to open SK3 channels, the proximity of both $Cav_{1.2}$ and SK3 channels to the SER in spines would support this hypothesis, with sufficient calcium release into the small spine cytosol to open large numbers of SK channels, based on data from other neurons showing that SK channels opening is detectable at ~0.2μM of calcium[26], which is within the range of calcium concentrations when released from the SER (~0.5-4μM)[27,28]. SK3 labeling was also seen in pyramidal-like dendritic shafts along the plasma membrane and trafficking on microtubules **(D-G**, where **D-E** are longitudinal, and **F-G** cross-sections of dendrites**)**, but showed limited axonal labeling. In **G**, the dendrite receives a symmetric Type II synapse from a putative GABAergic interneuron axon terminal (pseudo-colored purple). SK3 labeling is also seen in astrocytic processes **(C, F, G;** pseudocolored green**)**. Synapses are between arrows. Ax, axon; As, astroglial; Mit, mitochondria; Sp, dendritic spine; Den, dendrite; End, endosome. Scale bars, 200 nm.

© 2024 Datta D et al. *JAMA Psychiatry.*

eFigure 7

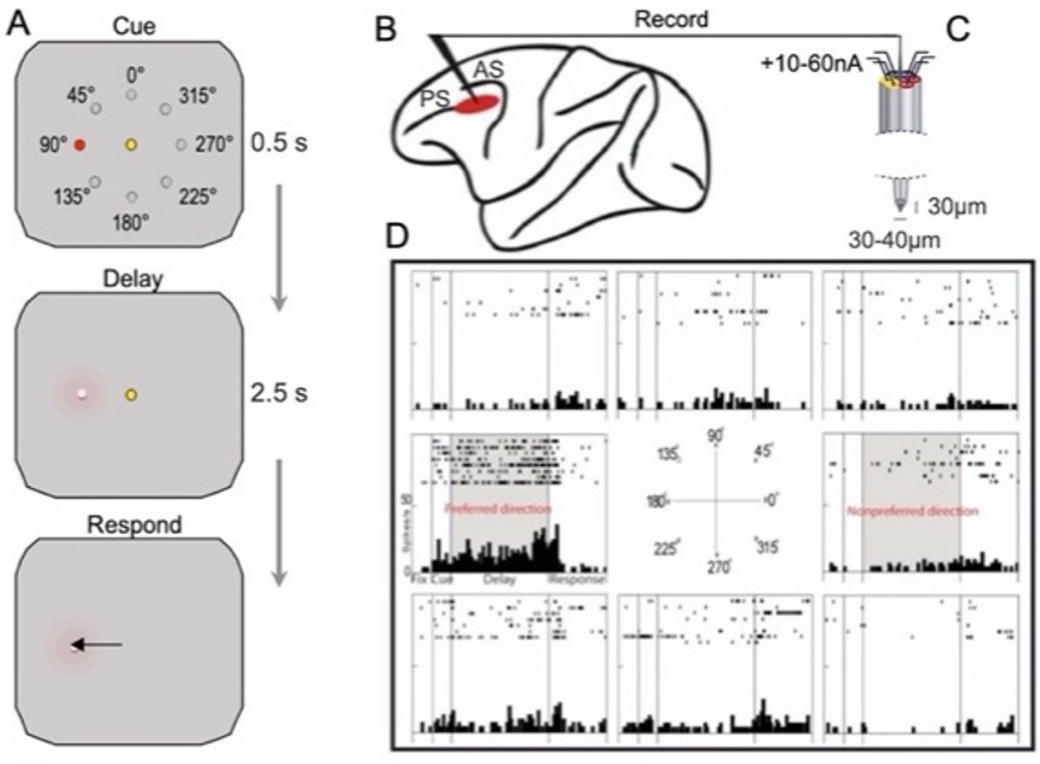

eFig. 7- Schematic of methods used for single unit recording from macaque dlPFC during a spatial working memory task, with iontophoretic delivery of drug to alter the local neurochemical environment. (A) Schematic of the ODR spatial working memory task, where the macaque initiates a trial by fixating on a central spot. A cue then appears for 0.5sec, followed by a delay period in which the monkey must remember the location of the cue for 2.5sec. At the end of the delay, the fixation point disappears and the monkey makes an eye movement to the remembered location for juice reward. The monkey can then fixate to initiate a new trial, where the cue appears randomly at one of the eight locations, and the contents of working memory must be updated with the new location. **(B)** The dlPFC recording site (PS=principal sulcus; AS=arcuate sulcus). **(C)** The iontophoretic electrode: a carbon fiber and surrounding glass micropipettes for drug delivery. **(D)** An example of a Delay cell, with sustained delay-related firing for its preferred direction, but not for nonpreferred directions. The degree of spatial tuning (firing for preferred vs. nonpreferred locations during the delay period) is quantified by signal detection using d'.

© 2024 Datta D et al. *JAMA Psychiatry.*

eFigure 8

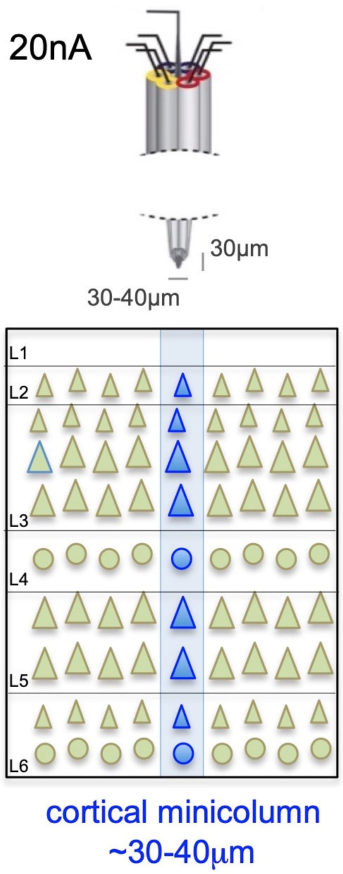

eFig. 8- Estimate of drug concentration from iontophoresis at 20nA at 100 µm from electrode tip. Iontophoresis delivers a very small amount of drug[29], insufficient to alter behavior, influencing a small region of cortex. The effects of iontophoresis are very localized, but the actual degree of spread is highly dependent on a large number of both known and unknown variables, including the electric charge of the compound, its lipophilicity, and the microenvironment of the electrode (e.g. degree of nearby myelin). Some attempts at estimates ex vivo have shown a diameter of about 30 µm, similar to a cortical minicolumn[30]. A rare *in vivo* experiment by Gerhardt and Palmer[29] performed an iontophoresis experiment where they locally applied dopamine from the iontophoretic electrode, and measured the dopamine levels 100µm away using electrochemical detection. The barrel concentration of dopamine was 0.5M, and a 20nA current produced an increase of ~17µM from baseline after 40 sec. The concentrations of drug used in the current study were lower (0.005-0.01M); thus, based on this previous work we would estimate that a 20nA current would produce a concentration of drug about 0.17-0.34µM at 100µm away from the electrode tip. However, the actual concentration of any specific compound cannot be known.

© 2024 Datta D et al. *JAMA Psychiatry.*

eFigure 9

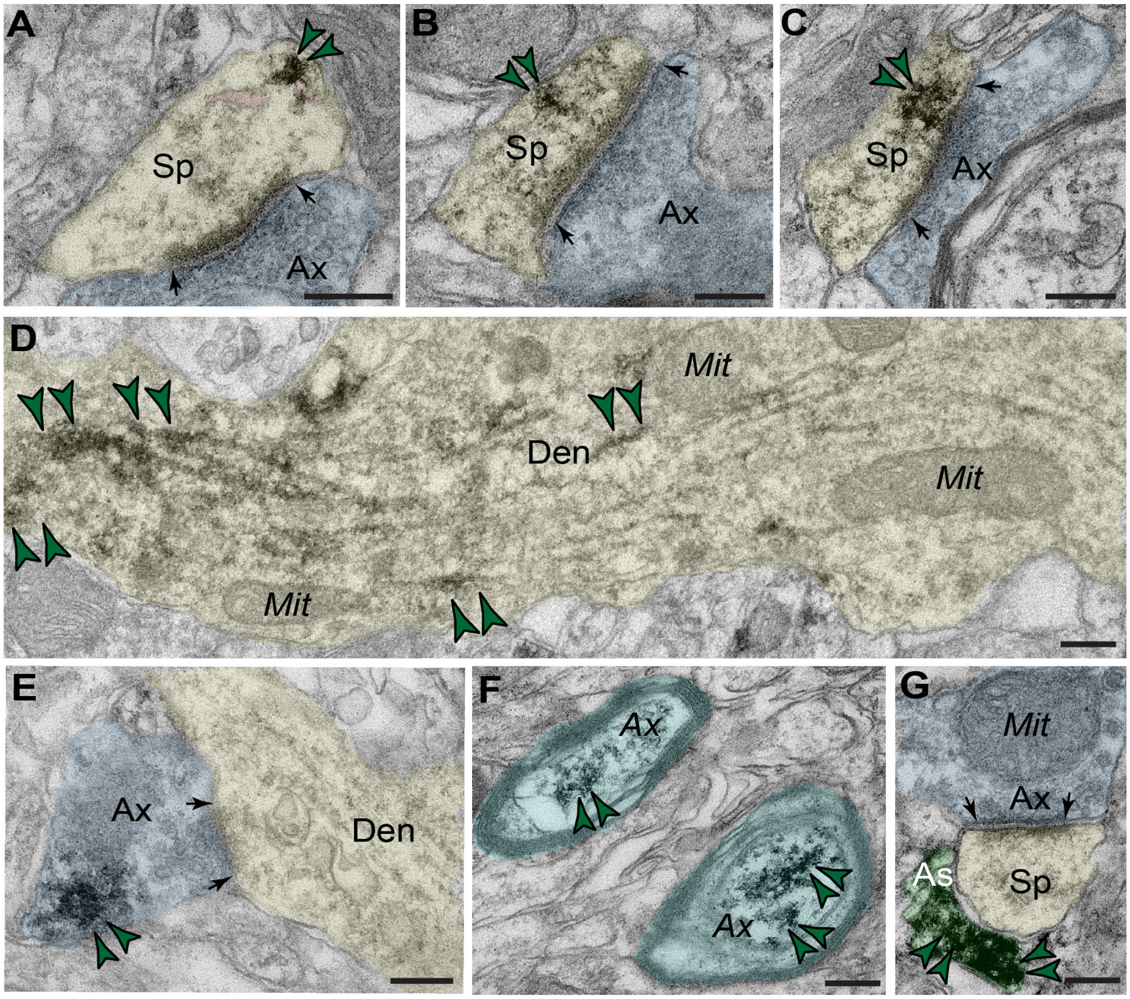

eFig. 9- Cav1.3 LTCCs have a more generalized expression pattern in macaque layer III dlPFC, including axonal labeling. (A-C) $Cav_{1.3}$ immunolabeling (dark teal arrowheads) can be seen in layer III dendritic spines, e.g. on the extrasynaptic plasma membrane **(A, B)** and under the post-synaptic density **(C)**. All dendritic spines receive axospinous Type I asymmetric glutamatergic-like synapses. **(D)** $Cav_{1.3}$ immunolabeling can be seen in layer III dendritic shafts with a pyramidal-like phenotype. This example of a longitudinal profile shows $Cav_{1.3}$ immunolabeling along microtubules, likely trafficking within the neuron. The localization in dendrites and spines is similar to $Cav_{1.2}$. **(E-F)** In contrast to $Cav_{1.2}$, $Cav_{1.3}$ immunolabeling is prominent in axons, including axon terminals **(E)**, and within myelinated axonal segments **(F)**. **(G)** $Cav_{1.3}$ immunolabeling is also seen in astrocytic leaflets ensheathing axospinous, asymmetric synapses. Panel **G** shows an example of $Cav_{1.3}$ immunolabeling in a likely astrocytic leaflet (green) near a glutamate-like synapse on a dendritic spine. The localization of $Cav_{1.3}$ (encoded by *CACNA1D*) on presynaptic axon terminals is consistent with gain-of-function mutations in *CACNA1D*, but not *CACNA1C*, being linked to increased risk of seizure disorders [31], as increased $Cav_{1.3}$ on axon terminals would increase glutamate release.
Synapses are between arrows. Ax, axon; As, astroglial; Mit, mitochondria; Sp, dendritic spine; Den, dendrite. Scale bars, 200 nm.

© 2024 Datta D et al. *JAMA Psychiatry.*

eFigure 10

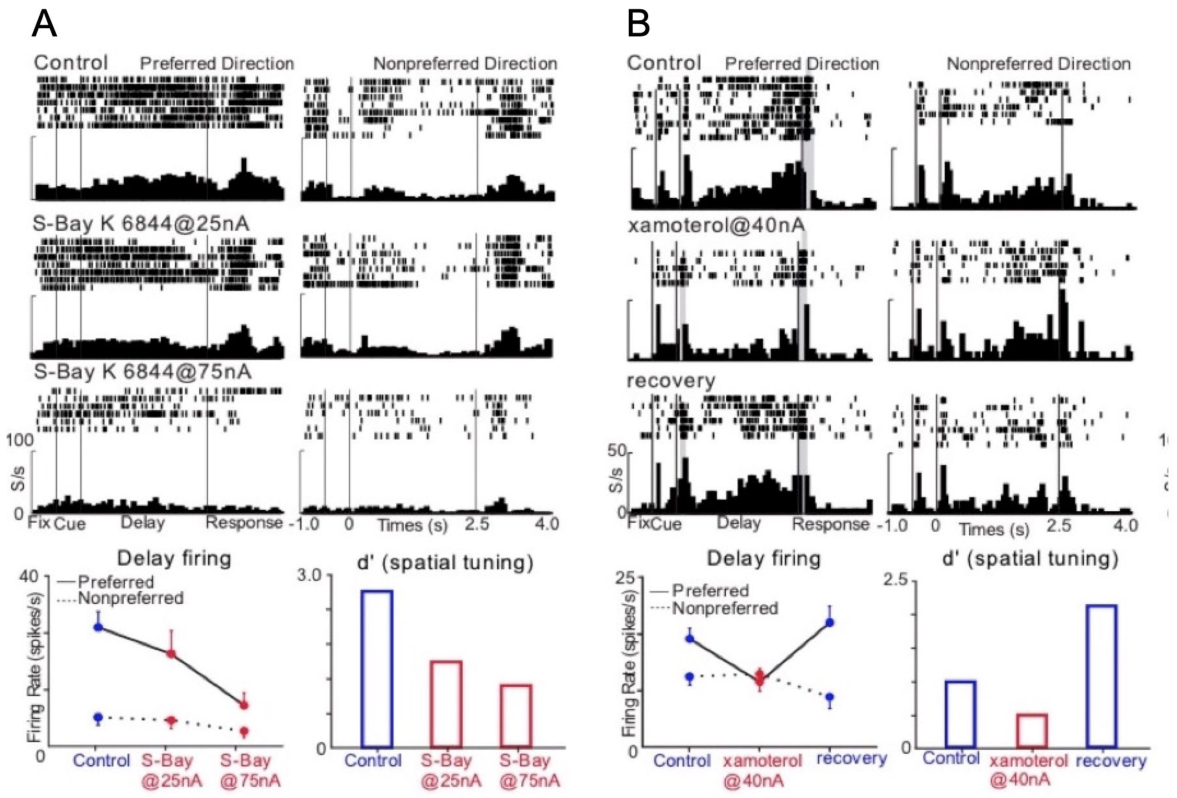

eFig. 10- Iontophoresis of the LTCC agonist, S-Bay K 6844, or the β1-AR agonist, xamoterol, markedly reduced delay-related firing and spatial tuning of dlPFC Delay cells.
(A) An example neuron (the same cell as Fig. 4E, left panel) is shown where iontophoresis of the L-type calcium channel (LTCC) activator, S-Bay K 6844, markedly reduced delay firing for the neuron's preferred direction. **Top:** Raster and histogram data shown for the preferred and nonpreferred directions. **Bottom left**: the mean±SEM firing rate of this cell during the Delay period of the task. S-Bay significantly reduced the delay-related firing for the preferred direction (repeated measures two-way ANOVA, $F_{directionxdrug}(2, 35) = 2.946$, p=0.0657; $F_{direction}(1, 35) = 30.44$, p<0.0001; $F_{drug}(2, 35) = 5.710$, p=0.0071; Tukey's multiple comparisons: preferred direction: control vs S-Bay@25nA, p=0.3409; control vs S-Bay@75nA, p=0.0009; S-Bay@25nA vs S-Bay@75nA, p=0.0316; nonpreferred direction, control vs S-Bay@25nA, p=0.9884; control vs S-Bay@75nA, p=0.7846; S-Bay@25nA vs S-Bay@75nA, p=0.8664). **Bottom right**: iontophoresis of S-Bay decreased the spatial tuning by decreasing d'. **(B)** An example neuron (the same cell as Fig. 5A left panel) showing that iontophoresis of the β1-AR agonist, xamoterol, markedly reduces delay firing for the neuron's preferred direction, and the firing was partially restored when xamoterol application was terminated (recovery). **Top:** Raster and histogram data shown for the preferred and nonpreferred directions. **Bottom left**: the mean±SEM firing rate of this cell during the Delay period of the task. Xamoterol significantly reduced the delay-related firing for the preferred direction (repeated measures two-way ANOVA, $F_{directionxdrug}(2, 35) = 5.854$, p=0.0064; $F_{direction}(1, 35) = 14.72$, p=0.0005; $F_{drug}(2, 35) = 2.093$, p=0.1385; Tukey's multiple comparisons: preferred direction: control vs xamo@40nA, p=0.0055; xamo@40nA vs recovery, p=0.002; control vs recovery, p=0.6591; nonpreferred direction, control vs xamo@40nA, p=0.9855; xamo@40nA vs recovery, p=0.446; control vs recovery, p=0.4342). **Bottom right**: Iontophoresis of xamoterol decreased the spatial tuning by decreasing d'.

© 2024 Datta D et al. *JAMA Psychiatry.*

eFigure 11

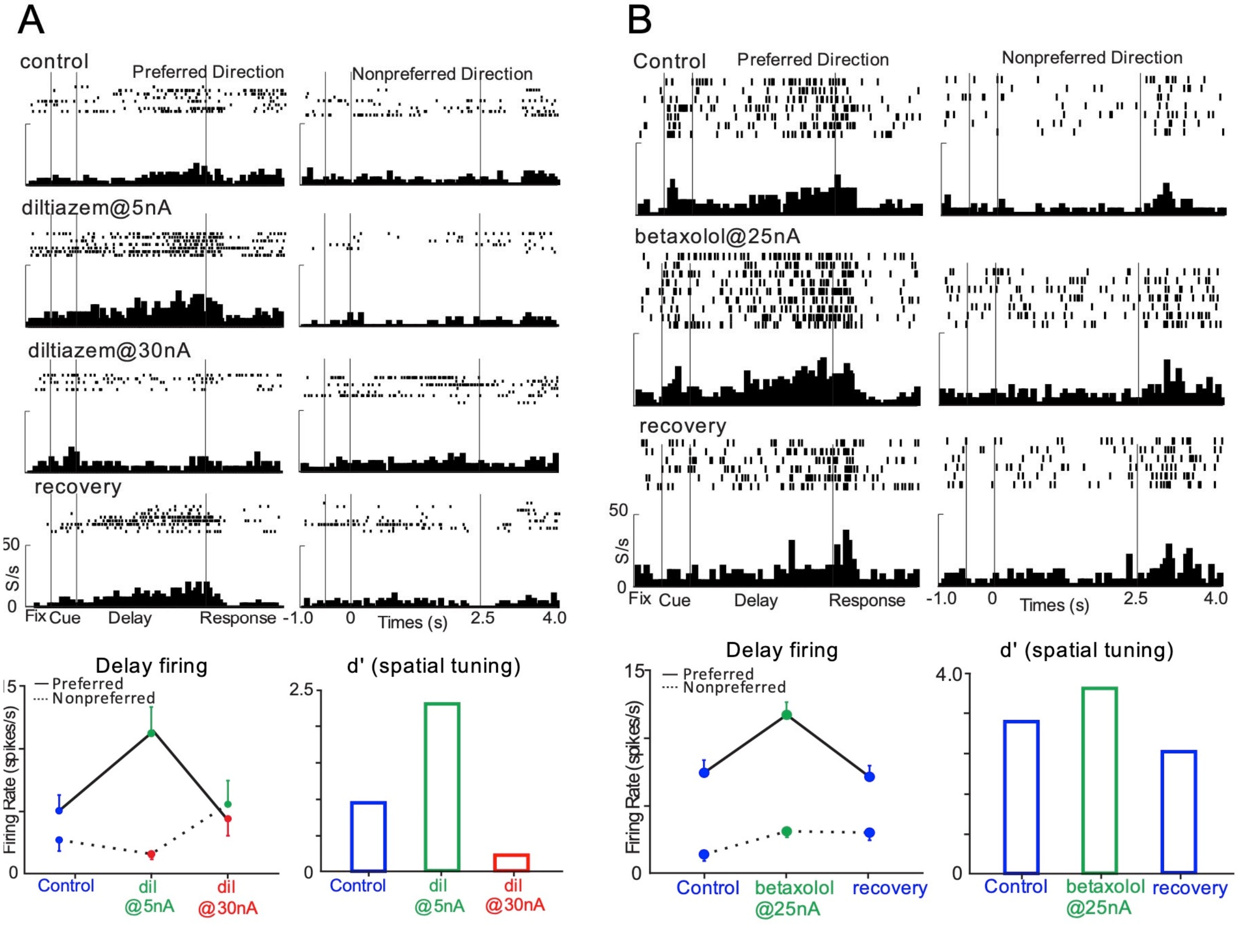

eFig. 11- Iontophoresis of the LTCC antagonist, diltiazem, produced an inverted-U dose response, while the β1-AR antagonist, betaxolol, markedly enhanced delay-related firing and spatial tuning of dlPFC Delay cells.

(A) An example neuron (the same cell as Fig. 4F, left panel) is shown where iontophoresis of the LTCC blocker, diltiazem, produced an inverted U effect on the delay firing. Low dose of diltiazem markedly enhanced delay firing for the neuron's preferred direction, while higher dose of diltiazem reduced the firing. **Top:** Raster and histogram data shown for the preferred and nonpreferred directions. **Bottom left**: the mean±SEM firing rate of this cell during the Delay period of the task. Iontophoresis of diltiazem, produced an inverted U effect on the delay firing (repeated measures two-way ANOVA, $F_{directionxdrug}$((3, 52) = 4.579, p=0.0064; $F_{direction}$(1, 52) = 14.11, p=0.0004; Fd_{rug}((3, 52) = 1.312, p=0.2805; Tukey's multiple comparisons: preferred direction: control vs dil@5nA, p=0.0294; control vs dil@30nA, p=0.9411; dil@5nA vs dil@30nA, p=0.0145; dil@30nA vs recovery, p=0.0719; non-preferred direction: control vs dil@5nA, p=0.9653; control vs dil@30nA, p=0.6205; dil@5nA vs dil@30nA, p=0.3905; dil@30nA vs recovery, p=0.619) **Bottom right**: Iontophoresis of diltiazem at 5nA increased the spatial tuning by increasing d', while iontophoresis of diltiazem at 30nA decreased the spatial tuning by decreasing d'. **(B)** An example neuron (the same cell as Fig. 5B, left panel) is shown where iontophoresis of the β1-AR antagonist, betaxolol, markedly enhanced delay firing for the neuron's preferred direction, and the firing was reduced to the control level when betaxolol

© 2024 Datta D et al. *JAMA Psychiatry.*

application was terminated (recovery). **Top:** Raster and histogram data shown for the preferred and nonpreferred directions. **Bottom left**: the mean±SEM firing rate of this cell during the Delay period of the task. Betaxolol significantly enhanced the delay-related firing for the preferred direction (repeated measures two-way ANOVA, $F_{directionxdrug}(2, 38) = 4.365$, p=0.0197; $F_{direction}(1, 38) = 99.55$, p<0.0001; $F_{drug}(2, 38) = 10.05$, p=0.0003; Tukey's multiple comparisons: preferred direction: control vs beta@25nA, p=0.0005; beta@25nA vs recovery, p=0.0004; control vs recovery, p=0.9634; nonpreferred direction, control vs beta@25nA, p=0.1746; beta@25nA vs recovery, p=0.9979; control vs recovery, p=0.3042). **Bottom right**: Iontophoresis of betaxolol increased the spatial tuning by increasing d'.

© 2024 Datta D et al. *JAMA Psychiatry.*

eFigure 12

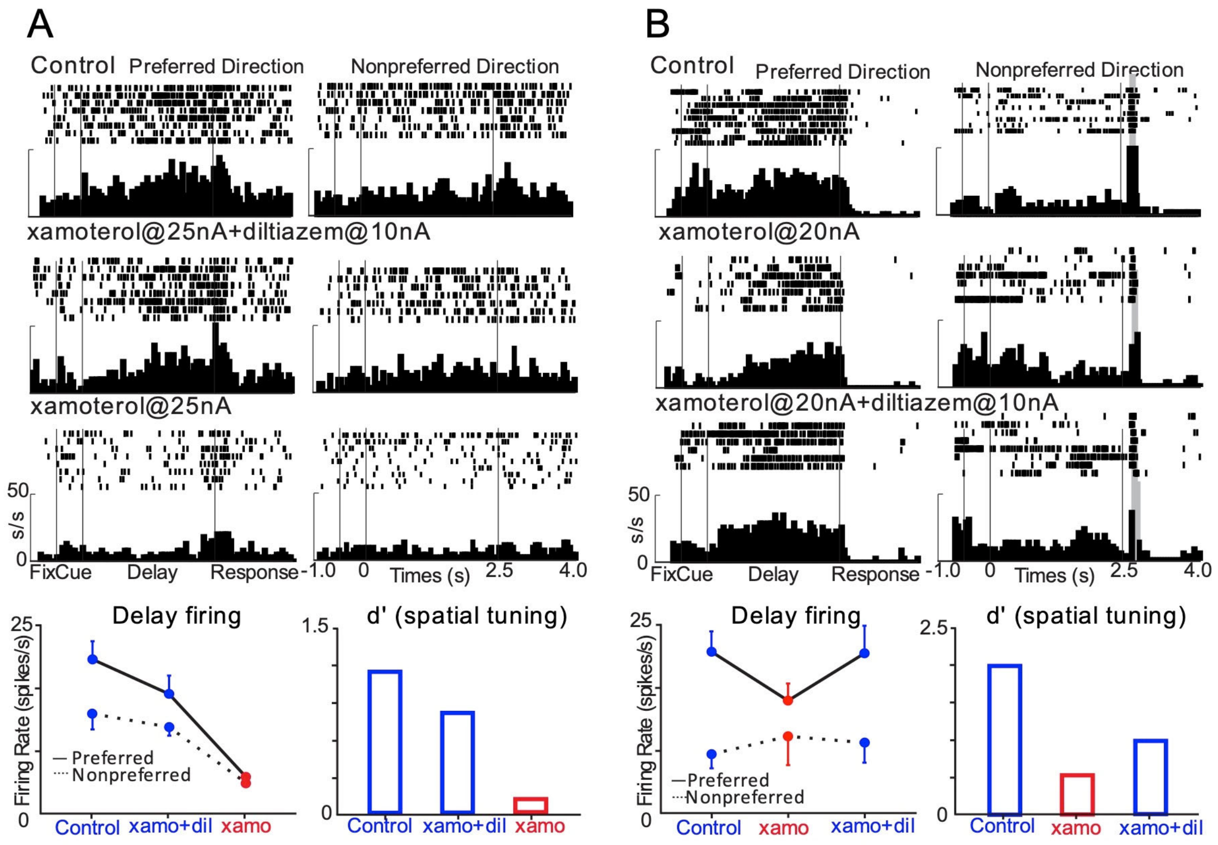

eFig. 12- Blocking LTCCs prevents/reverses the reduction in firing from β1-AR stimulation. **(A)** An example neuron (the same cell as Fig. 5C, left panel) is shown where iontophoresis of the LTCC blocker, diltiazem, prevented the reducing effects of the β1-AR agonist, xamoterol, when these two agents were co-applied. **Top:** Raster and histogram data shown for the preferred and nonpreferred directions. **Bottom left**: the mean±SEM firing rate of this cell during the Delay period of the task. Co-application of diltiazem with xamoterol prevented the delay firing reduction caused by xamoterol (repeated measures two-way ANOVA, $F_{directionxdrug}(2, 41) = 2.120$, p=0.1330; $F_{direction}(1, 41) = 7.110$, p=0.0109; $F_{drug}(2, 41) = 27.25$, p<0.0001; Tukey's multiple comparisons: preferred direction: control vs dil+xamo, p=0.1651; control vs xamo, p<0.0001; dil+xamo vs xamo, p=0.0001; non-preferred direction: control vs dil+xamo, p=0.7833; control vs xamo, p<0.0029; dil+xamo vs xamo, p=0.0196). **Bottom right**: Co-application of diltiazem with xamoterol prevented the spatial tuning reduction caused by xamoterol. **(B)** An example neuron (Carl339u14) is shown where iontophoresis of the LTCC blocker, diltiazem, rescued the reduction in firing caused by the β1-AR agonist, xamoterol, when these two agents were co-applied. **Top:** Raster and histogram data shown for the preferred and nonpreferred directions. **Bottom left**: the mean±SEM firing rate of this cell during the Delay period of the task. Co-application of diltiazem with xamoterol rescued the delay firing reduction caused by xamoterol (repeated measures two-way ANOVA, $F_{directionxdrug}(1, 27) = 4.745$, p=0.0383; $F_{direction}(1, 27) = 12.20$, p=0.0017; $F_{drug}(1, 27) = 1.566$, p=0.2215; Sidak's multiple comparisons: control vs xamo: preferred direction: p=0.0409; non-preferred direction: p=0.7726). **Bottom right**: Co-application of diltiazem with xamoterol reversed the spatial tuning reduction caused by xamoterol.

© 2024 Datta D et al. *JAMA Psychiatry.*

eFigure 13

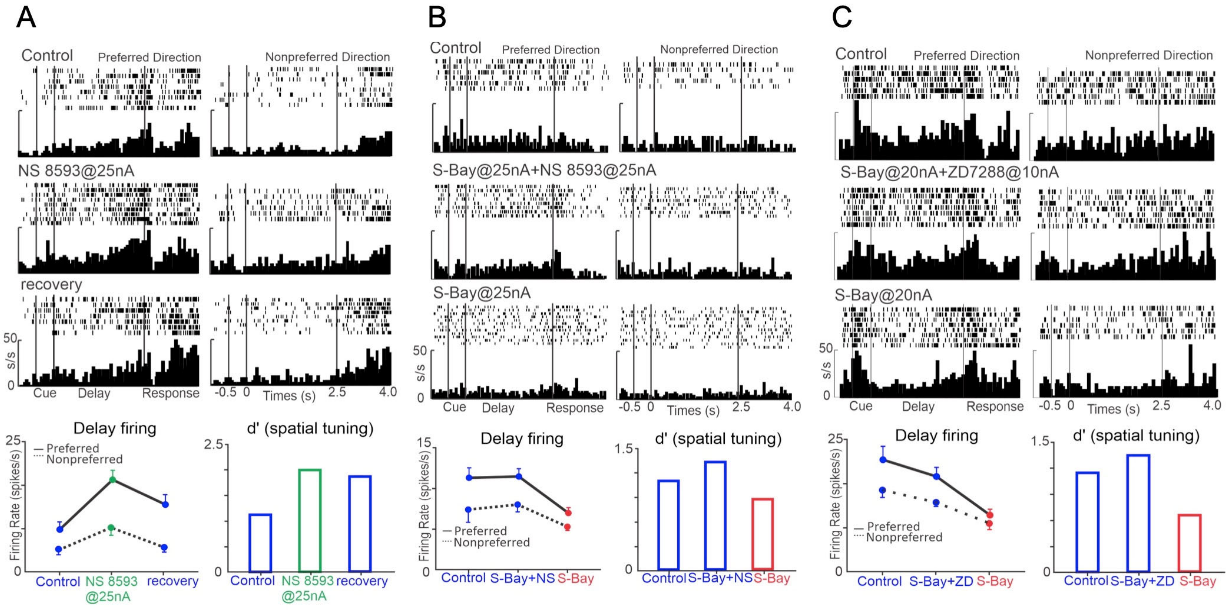

eFig. 13- Iontophoresis of the SK channel blocker, NS8593, markedly enhanced the delay-related firing and spatial tuning of dlPFC Delay cells, while blockade of either SK or HCN channels blocked the effects of LTCC opening. (A) An example neuron (the same cell as Fig. 6A, left panel) is shown where iontophoresis of the SK channel blocker, NS8593, markedly enhanced delay firing for the neuron's preferred direction, and the firing was reduced to control levels when NS8593 application was terminated (recovery). **Top:** Raster and histogram data shown for the preferred and nonpreferred directions. **Bottom left**. The mean±SEM firing rate of this cell during the Delay period of the task. NS8593 significantly enhanced the delay-related firing for the preferred direction (repeated measures two-way ANOVA, $F_{directionxdrug}(2, 40) = 1.773$, p=0.1829; $F_{direction}(1, 40) = 32.14$, p<0.0001; $F_{drug}(2, 40) = 9.993$, p=0.0003; Tukey's multiple comparisons: preferred direction: control vs NS@25nA, p<0.0001; NS@25nA vs recovery, p=0.0668; control vs recovery, p=0.0855; nonpreferred direction, control vs NS @25nA, p=0.1959; NS @25nA vs recovery, p=0.2306; control vs recovery, p=0.978). **Bottom right**. Iontophoresis of NS8593 increased the spatial tuning as indicated by an increase in d'. **(B)** An example neuron (the same cell as Fig. 6B, left panel) is shown where iontophoresis of the SK blocker, NS8593, prevented the reduction in firing caused by the LTCC activator, S-Bay, when these two agents were co-applied. **Top:** Raster and histogram data shown for the preferred and nonpreferred directions. **Bottom left**. The mean±SEM firing rate of this cell during the Delay period of the task. Co-application of NS8593 with S-Bay prevented the delay firing reduction caused by S-Bay (repeated measures two-way ANOVA, $F_{directionxdrug}(2, 39) = 1.091$, p=0.3459; $F_{direction}(1, 39) = 13.81$, p=0.0006; $F_{drug}(2, 39) = 9.020$, p=0.0006; Tukey's multiple comparisons: preferred direction: control vs NS+S-Bay, p=0.979; control vs S-Bay, p=0.0082; NS+S-Bay vs S-Bay, p=0.0021; non-preferred direction: control vs NS+S-Bay, p=0.9111; control vs S-Bay, p=0.4288; NS+S-Bay vs S-Bay, p=0.1456). **Bottom right**. Co-application of NS8593 with S-Bay prevented the spatial tuning reduction caused by S-Bay. **(C)** An example neuron (the same cell as Fig. 6C, left panel) is shown where iontophoresis of the HCN blocker, ZD7288, prevented the reduction in firing caused by the LTCC activator, S-Bay, when these two agents were co-applied. **Top:** Raster and histogram data shown for the preferred and nonpreferred directions. **Bottom**

© 2024 Datta D et al. *JAMA Psychiatry.*

left. The mean±SEM firing rate of this cell during the Delay period of the task. Co-application of ZD7288 with S-Bay prevented the delay firing reduction caused by S-Bay (repeated measures two-way ANOVA, $F_{directionxdrug}(2, 34) = 0.8820$, p=0.4232; $F_{direction}(1, 34) = 10.39$, p=0.0028; $F_{drug}(2, 34) = 14.68$, p<0.0001; Tukey's multiple comparisons: preferred direction: control vs ZD+S-Bay, p=0.1654; control vs S-Bay, p=0.0001; ZD+S-Bay vs S-Bay, p=0.0065; non-preferred direction: control vs ZD+S-Bay, p=0.5167; control vs S-Bay, p=0.0194; ZD+S-Bay vs S-Bay, p=0.1666). **Bottom right**. Co-application of ZD7288 with S-Bay prevented the spatial tuning reduction caused by S-Bay.

© 2024 Datta D et al. *JAMA Psychiatry.*

eFigure 14

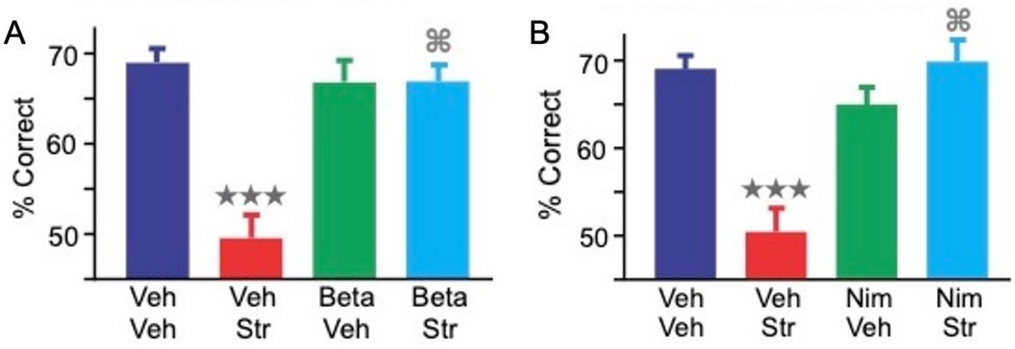

eFig. 14- Behavioral data showing performance of the spatial delayed response test of spatial working memory under control vs. stress conditions, with pretreatment with vehicle or test drug. A dose of the β1-AR antagonist, betaxolol (between 0.001-0.1 mg/kg, p.o., one hour before testing) **(A)** or of the LTCC blocker, nimodipine (0.1-1.0 mg/kg, p.o., **(B)** one hour before testing) was identified for each animal that did not improve performance on its own, to challenge the stress response without concerns of additive effects. The nimodipine dose was similar to that used in humans. Pretreatment with systemic administration of a β1-AR antagonist or an LTCC antagonist protected working memory performance from the detrimental effects of a mild stressor. Vehicle (Veh) pretreatment in combination with the pharmacological stressor, FG7142 (Str) significantly impaired performance compared to vehicle+vehicle control performance, while pretreatment with the β1-AR antagonist betaxolol (Beta) or the LTCC antagonist, nimodipine (Nim), prevented working memory deficits in FG7142-treated animals. Results represent mean percent correct on the delayed response task with all individual data points shown (n=6). *** $p \leq 0.001$ compared to veh+veh; ⌘ $p<0.001$ compared to veh+stress.

© 2024 Datta D et al. *JAMA Psychiatry.*

eFigure 15

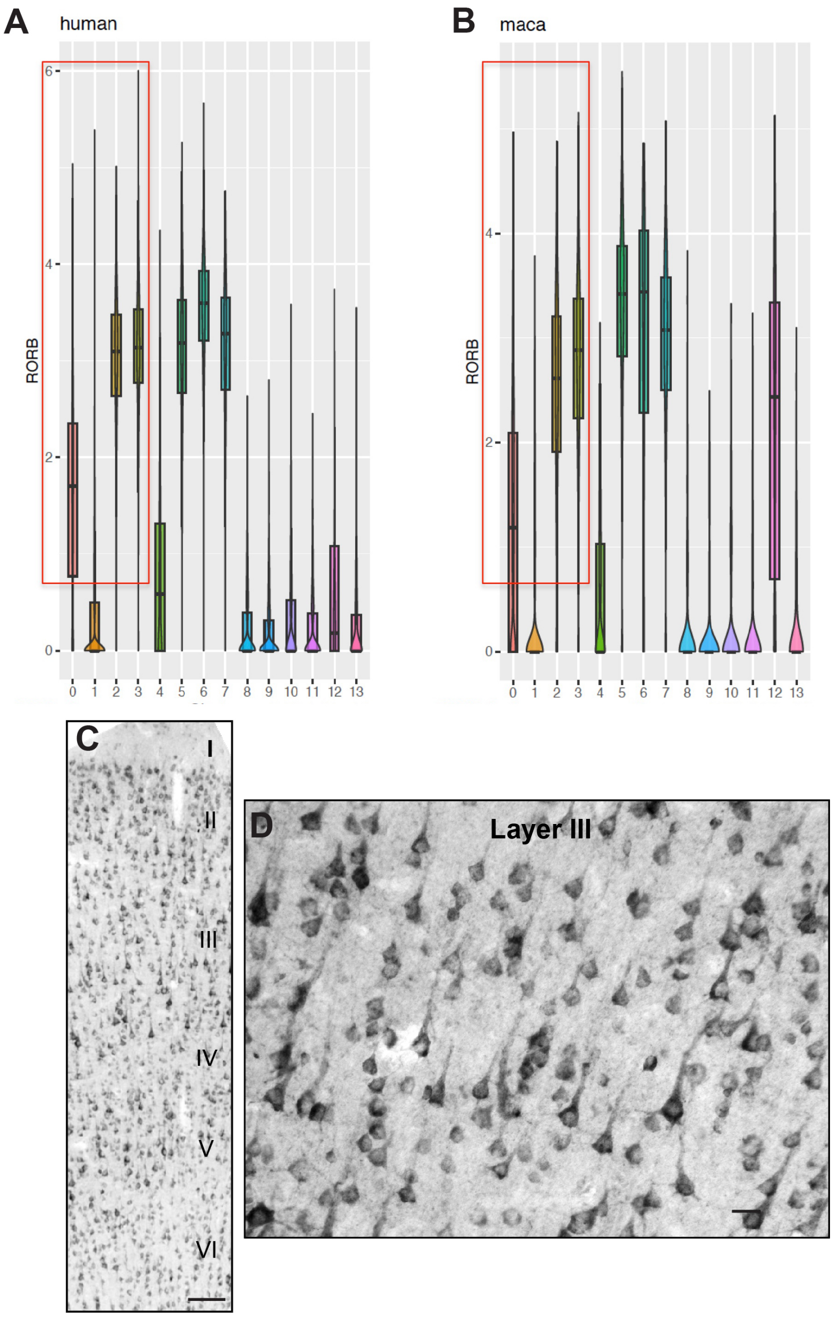

eFig. 15- *RORB* is widely expressed in dlPFC pyramidal cells, including in the CUX2A and CUX2C subgroups. **(A)** Violin plot of *RORB* expression in human dlPFC pyramidal cells. **(B)** Violin plot of *RORB* expression in macaque dlPFC pyramidal cells. The CUX2A cell group is Cluster 0, the CUX2B cell group is Cluster 1, the CUX2C cell group is Cluster 3, and the PLD5A cell group is Cluster 11. **(C)** Widespread expression of RORB in macaque dlPFC across layers, including high expression in many layer III pyramidal cells **(D)**. These protein results for RORB are corroborated by recent RNA-seq results in macaque[32] and human[33] dlPFC layer III pyramidal cells using laser-capture microdissection methods, providing laminar- and cell-type specific resolution.

© 2024 Datta D et al. *JAMA Psychiatry.*

eReferences

1. González-Burgos G, Miyamae T, Krimer Y, et al. Distinct Properties of Layer 3 Pyramidal Neurons from Prefrontal and Parietal Areas of the Monkey Neocortex. *J Neurosci*. 2019;39:7277-90.
2. Saunders A, Macosko EZ, Wysoker A, et al. Molecular Diversity and Specializations among the Cells of the Adult Mouse Brain. *Cell*. 2018;174:1015-30.
3. Datta D, Leslie S, Wang M, et al. Age-related calcium dysregulation linked with tau pathology and impaired cognition in non-human primates. *Alzheimer's & Dementia*. 2021;17:920-32.
4. Jeon D, Kim S, Chetana M, et al. Observational fear learning involves affective pain system and Cav1.2 Ca2+ channels in ACC. *Nat Neurosci* 2010;13:482-8.
5. Kim S, Ma L, Jensen KL, et al. Paradoxical contribution of SK3 and GIRK channels to the activation of mouse vomeronasal organ. *Nat Neurosci* 2012;15:1236-44.
6. Shi L, Ko ML, Ko GY. Retinoschisin Facilitates the Function of L-Type Voltage-Gated Calcium Channels. *Front Cell Neurosci* 2017;11:232.
7. Lee M, Mueller A, Moore T. Differences in Noradrenaline Receptor Expression Across Different Neuronal Subtypes in Macaque Frontal Eye Field. *Front Neuroanat*. 2020;14:574130. doi:https://doi.org/10.3389/fnana.2020.574130
8. Mukherjee B, Yuan Q. NMDA receptors in mouse anterior piriform cortex initialize early odor preference learning and L-type calcium channels engage for long-term memory. . *Sci Rep*. 2016;6:35256.
9. Philippart F, Destreel G, Merino-Sepúlveda P, Henny P, Engel D, Seutin V. Differential Somatic Ca2+ Channel Profile in Midbrain Dopaminergic Neurons. *J Neurosci*. 2016;36:7234-45.
10. Tu H, Liu J, Zhang D, et al. Heart failure-induced changes of voltage-gated Ca2+ channels and cell excitability in rat cardiac postganglionic neurons. *Am J Physiol Cell Physiol*. 2014;306:C132-42.
11. Yap FC, Weber DS, Taylor MS, et al. Endothelial SK3 channel-associated Ca2+ microdomains modulate blood pressure. *Am J Physiol Heart Circ Physiol*. 2016;310:H1151-63.
12. Fiala JC. Reconstruct: a free editor for serial section microscopy. *J Microsc*. 2005;218:52-61.
13. Goldman-Rakic P. Cellular Basis of Working Memory. *Neuron*. 1995 1995;14:477-485.
14. Funahashi S, Bruce CJ, Goldman-Rakic PS. Dorsolateral prefrontal lesions and oculomotor delayed-response performance: evidence for mnemonic "scotomas". *J Neurosci*. 1993;13:1479-97.
15. Chafee MV, Goldman-Rakic PS. Inactivation of parietal and prefrontal cortex reveals interdependence of neural activity during memory-guided saccades. *J Neurophysiol* 2000;83:1550-66.
16. Suzuki M, Gottlieb J. Distinct neural mechanisms of distractor suppression in the frontal and parietal lobe. *Nat Neurosci*. 2013;16:98-104.
17. Bastos AM, Loonis R, Kornblith S, Lundqvist M, Miller EK. Laminar recordings in frontal cortex suggest distinct layers for maintenance and control of working memory. *Proc Natl Acad Sci U S A*. 2018;115:1117-22.
18. Constantinidis C, Funahashi S, Lee D, et al. Persistent Spiking Activity Underlies Working Memory. *J Neurosci* 2018;38:7020-8.
19. Wang M, Yang Y, Wang CJ, et al. NMDA receptors subserve working memory persistent neuronal firing In dorsolateral prefrontal cortex. *Neuron*. 2013;77(4):736-49.
20. Dorow R, Horowski R, Pashelke G, Amin M, Braestrup C. Severe anxiety induced by FG7142, a B-carboline ligand for benzodiazepine receptors. *The Lancet*. 1983;2:98-99.

© 2024 Datta D et al. *JAMA Psychiatry.*

21. Ninan PT, Insel TM, Cohen RM, Cook JM, Skolnick P, Paul SM. Benzodiazepine receptor-mediated experimental anxiety in primates. *Science*. 1982;218(4579):1332-4.
22. Takamatsu H, Noda A, Kurumaji A, et al. A PET study following treatment with a pharmacological stressor, FG7142, in conscious rhesus monkeys. *Brain Res*. 2003;980:275-80.
23. Leidenheimer NJ, Schechter MD. Discriminative stimulus control by the anxiogenic b-carboline FG7142: Generalization to a physiological stressor. *Pharmacology Biochemistry Behavior*. 1988;30:351-355.
24. Mikkelsen JD, Søderman A, Kiss A, Mirza N. Effects of benzodiazepines receptor agonists on the hypothalamic-pituitary-adrenocortical axis. *Eur J Pharmacol*. 2005;519:223-30.
25. Dazzi L, Vignone V, Seu E, Ladu S, Vacca G, Biggio G. Inhibition by venlafaxine of the increase in norepinephrine output in rat prefrontal cortex elicited by acute stress or by the anxiogenic drug FG 7142. *J Psychopharmacol*. 2002;16:125-131.
26. Hirschberg B, Maylie J, Adelman JP, Marrion NV. Gating of recombinant small-conductance Ca-activated K+ channels by calcium. *J Gen Physiol*. 1998;111:565-81.
27. Usachev Y, Shmigol A, Pronchuk N, Kostyuk P, Verkhratsky A. Caffeine-induced calcium release from internal stores in cultured rat sensory neurons. *Neuroscience*. 1993;57:845-59.
28. Moshkforoush A, Ashenagar B, Tsoukias NM, Alevriadou BR. Modeling the role of endoplasmic reticulum-mitochondria microdomains in calcium dynamics. *Sci Rep*. 2019;9:17072.
29. Gerhardt GA, Palmer MR. Characterization of the techniques of pressure ejection and microiontophoresis using in vivo electrochemistry. *J Neurosci Methods*. 1987;22:147-59.
30. Odackal J, Colbourn R, Odackal NJ, Tao L, Nicholson C, Hrabetova S. Real-time Iontophoresis with Tetramethylammonium to Quantify Volume Fraction and Tortuosity of Brain Extracellular Space. *J Vis Exp*. 2017;125:55755.
31. Pinggera A, Mackenroth L, Rump A, et al. New gain-of-function mutation shows CACNA1D as recurrently mutated gene in autism spectrum disorders and epilepsy. *Hum Mol Genet*. 2017;26:2923-32.
32. Arion D, Enwright JF, Gonzalez-Burgos G, Lewis DA. Differential gene expression between callosal and ipsilateral projection neurons in the monkey dorsolateral prefrontal and posterior parietal cortices. *Cereb Cortex*. 2023;33:1581-94.
33. Enwright Iii JF, Arion D, MacDonald WA, et al. Differential gene expression in layer 3 pyramidal neurons across 3 regions of the human cortical visual spatial working memory network. *Cereb Cortex*. Nov 9 2022;32(22):5216-5229. doi:10.1093/cercor/bhac009

© 2024 Datta D et al. *JAMA Psychiatry.*
