# Supplementary material for: Key Roles of CACNA1C/Cav1.2 and CALB1/Calbindin in Prefrontal Neurons Altered in Cognitive Disorders
Source: JAMA Psychiatry. 2024 May 22;81(9):870–81. doi: 10.1001/jamapsychiatry.2024.1112 (PMC11112502; doi:10.1001/jamapsychiatry.2024.1112)
Supplement: Supplement 3. — Data sharing statement [file jamapsychiatry-e241112-s003.pdf]

Data Sharing Statement

Datta. Key Roles of CACNA1C/Cav1.2 and CALB1/Calbindin in Prefrontal Neurons Altered in Cognitive Disorders. *JAMA Psychiatry*. Published May 22, 2024.
doi:10.1001/jamapsychiatry.2024.1112

Data
Data available: No

Additional Information
Explanation for why data not available: All of the data are in the paper already Note this is not a clinical trial or a true genetic association study, but rather human and macaque transcriptomics, and macaque anatomy, physiology and behavior examining mechanism
